# DNA binding activity of the proximal C-terminal domain of rat DNA topoisomerase IIβ is involved in ICRF-193-induced closed-clamp formation

**DOI:** 10.1371/journal.pone.0239466

**Published:** 2020-09-22

**Authors:** Shinji Kawano, Kunpei Fujimoto, Kazushi Yasuda, Shogo Ikeda

**Affiliations:** Department of Biochemistry, Faculty of Science, Okayama University of Science, Okayama, Japan; Saint Louis University, UNITED STATES

## Abstract

DNA topoisomerase II (topo II) is an essential enzyme that regulates DNA topology by DNA cleavage and re-ligation. In vertebrates, there are two isozymes, α and β. The C-terminal domain (CTD) of the isozymes, which shows a low degree of sequence homology between α and β, is involved in each isozyme-specific intracellular behavior. The CTD of topo IIβ is supposedly involved in topo II regulation. Topo IIβ is maintained in an inactive state in the nucleoli by the binding of RNA to the 50-residue region termed C-terminal regulatory domain (CRD) present in the CTD. Although *in vitro* biochemical analysis indicates that the CTD of topo IIβ has DNA binding activity, it is unclear whether CTD influences catalytic reaction in the nucleoplasm. Here, we show that the proximal CTD (hereafter referred to as pCTD) of rat topo IIβ, including the CRD, is involved in the catalytic reaction in the nucleoplasm. We identified the pCTD as a domain with DNA binding activity by *in vitro* catenation assay and electrophoretic mobility shift assay. Fluorescence recovery after photo-bleaching (FRAP) analysis of pCTD-lacking mutant (ΔpCTD) showed higher mobility in nucleoplasm than that of the wild-type enzyme, indicating that the pCTD also affected the nuclear dynamics of topo IIβ. ICRF-193, one of the topo II catalytic inhibitors, induces the formation of closed-clamp intermediates of topo II. Treatment of ΔpCTD with ICRF-193 significantly decreased the efficiency of closed-clamp formation. Altogether, our data indicate that the binding of topo IIβ to DNA through the pCTD is required for the catalytic reaction in the nucleoplasm.

## Introduction

DNA topoisomerase II (topo II) is an enzyme that catalyzes the cleavage and re-ligation of double-stranded DNA in the presence of ATP, thereby regulating DNA topology and resolving the DNA supercoiling that occurs during transcription, replication, and chromosome segregation [1]. Topo II is conserved from prokaryotes to eukaryotes and consists of three structural domains: an N-terminal domain with ATPase activity (NTD), a catalytic core domain, and a C-terminal domain (CTD). In eukaryotes, the functional enzyme is a homodimer. The structures of the NTD and catalytic core domain of the topo II dimer excluding CTD have been revealed by x-ray crystallography [2–4]. The eukaryotic CTDs appear to be structurally disordered and have not yet been analyzed by crystal structure analysis. A major role of the CTD in eukaryotic topo II is nuclear localization. Studies with CTD deletion mutants have shown that lack of CTD does not affect *in vitro* catalytic activity but lacks intracellular function [5–7].

In vertebrates, α and β isozymes are encoded in genomic DNA as independent genes [8–10]. The homology of the amino acid sequences in the regions of NTD and catalytic core domain is as high as about 70% between topo IIα and β, but CTD shows more sequence diversity. The diversity of CTD between topo IIα and β is thought to be related to the difference in the function of the isozymes.

Expression of topo IIα is cell cycle-dependent in proliferating cells [11]. Analysis of mutants in which the CTDs of topo IIα and β were swapped revealed that the CTD of topo IIα is required for mitotic chromosome localization [12]. Besides, chromatin tether (ChT) domain has been identified at the end of topo IIα CTD that allows binding to mitotic chromatin through the recognition of histone tail post-translational modifications [13]. Furthermore, *in vitro* biochemical studies have shown that the CTD of topo IIα affects the DNA cleavage activity, catalytic preference for positive supercoiled DNA, and chiral discrimination [14–16].

Topo IIβ is essential for the terminal differentiation of neuronal cells by regulating gene expression [17, 18]. Topo IIβ is not essential for cell proliferation, but it has been reported that topo IIβ-mediated double-strand break is formed in promoters of genes stimulated by hormone [19]. It has been shown that in differentiating neuronal cells, most of the topo IIβ localize to the nucleoplasm and is involved in catalysis, whereas in mature neuronal cells, the enzyme is concentrated in nucleoli and has less access to chromatin DNA [17, 20]. The nuclear distribution of topo IIβ reflects its enzymatic activity; moreover, in the nucleolus, the enzyme is maintained in an inactive state by the interaction of the 50-amino acid region (CRD) identified in the CTD of topo IIβ with RNA [21]. Although *in vitro* DNA-binding studies have shown that the CTD of topo IIβ has DNA-binding and DNA retention activities [21], it remains unclear how the CTD is involved in catalytic reaction in the nucleoplasm.

In this study, we aimed to identify a region in the CTD of topo IIβ involved in DNA binding and to clarify the effect of the CTD DNA binding activity on the catalytic reaction in the nucleoplasm. We identified the proximal CTD, which includes the CRD, as a region affecting the catalytic reaction of rat topo IIβ.

## Material and methods

### Plasmids

The plasmids (pFLAG-top2b and pFLAG-top2b-EGFP) constructed in a previous study [21] were used for the expression of full-length rat topo IIβ (WT), and CTD truncated mutants (ΔCTD, ΔCRD, and ΔCTD’). Expression vectors (1–1320 and Δ1201–1320) were constructed by cloning the PCR products of 1201–1320 and 1321–1614 into the ΔCTD expression vector, respectively. The primer sequences are shown in S1 Table. The I865A and Y814S mutants of topo IIβ were constructed by site-directed mutagenesis using KOD-Plus-Mutagenesis Kit (Toyobo). The primer sets used to generate the mutants were: for I865A, 5′- GCT GGT ACC GGA TGG GCT TGT AAA TTG CCC AAC -3′ and 5′- ACC CTC AGC ACC ATT AAT CAA AAC CAT GGG GAT T -3′; for Y814S, 5′- CG ATC TTC A CA ATG TTA AGC TCT CTG GCA A -3′ and 5′- A TCG GGG GCT TGC AGC ATC TTT GCC ACC GT -3′. GST-tagged recombinant protein expression vectors for rat topo IIb (1201–1614, 1201–1320, and 1321–1614) were constructed by inserting the PCR products into pGEX-6P vectors (GE Healthcare). The primer sequences are shown in S1 Table. The sequences of all constructs were verified using a BigDye Terminator v3.1 Cycle Sequencing Kit (Applied Biosystems).

### Cell culture and transfection

HEK293 cells (ATCC) were grown at 37°C in a humidified atmosphere of 5% CO_2_ in a Dulbecco’s modified Eagle medium (DMEM; Nissui) supplemented with 10% fetal bovine serum (FBS) and 100 μg/mL kanamycin sulfate. For protein purification, HEK293 cells were seeded in 6 well plates. For fluorescence imaging, HEK293 cells were grown in glass bottom dishes. Transfection was performed with FuGENE6 (Promega), following the manufacturer’s instructions.

### Protein expression and purification

FLAG-tagged proteins were expressed in HEK293 cells and purified as previously described [22]. The proteins were immunoprecipitated with anti-FLAG M2 (Sigma) immobilized on Dynabeads Protein G (Veritas) and purified from the beads using 3× FLAG peptide (Sigma). Purified proteins were frozen in liquid N_2_ and stored at -80°C until use. GST-tagged proteins were expressed in *E*. *coli* strain JM109 (Nippon Gene) and purified as previously described [22].

### *In vitro* catalytic assay

Relaxation and catenation assays were performed, as described in Kawano *et al*. [22]. For the relaxation assay, negative supercoiled plasmid pUC18 DNA was incubated at 30°C for 30 min in 10 μL topo II reaction buffer (50 mM Tris-HCl pH 8.0, 120 mM KCl, 10 mM MgCl_2_, 0.5 mM EDTA, 1 mM DTT, 0.5 mM ATP, and 30 μg/mL bovine serum albumin) with FLAG-tagged topo IIβ. The reaction was terminated by adding 1% SDS and 0.2 μg/μL proteinase K (Roche). After incubation at 55°C for 1 h, the reaction products were analyzed by agarose gel electrophoresis. DNA bands were detected using GelRed Nucleic Acid Gel Stain (Biotium) or SYBR Green I (Takara Bio). The catenation assay was performed as described for the relaxation assay, except that histone H1.0 (NEB) and PEG 8000 (Sigma) were used as DNA condensing agents. Decatenation assays was carried out as described for the relaxation assay, except that a kinetoplast DNA (Inspiralis) was used as substrate.

### Electrophoretic mobility shift assay (EMSA)

EMSA was carried out using a previously described method [22]. GST-tagged recombinant proteins were mixed with 5 ng supercoiled and linearized pUC18 each in 10 μL binding buffer (50 mM Tris-HCl pH 8.0, 120 mM KCl, 10 mM MgCl_2_, 0.5 mM EDTA, and 30 μg/mL bovine serum albumin). After incubation at 30°C for 30 min, the reaction mixtures were immediately separated on 1% agarose gels 10 mM MgCl_2_. Tris-borate-EDTA buffer (0.5× concentration) was used for the running buffer. DNA bands were detected by staining with GelRed Nucleic Acid Gel Stain.

### DNA cleavage assay

DNA cleavage assays were performed by incubating 5 ng pUC18 with FLAG-tagged topo IIβ (50, 100, and 200 fmol) in 10 μL cleavage buffer (50 mM Tris-HCl pH 8.0, 120 mM KCl, 10 mM MgCl_2_, 0.5 mM EDTA, 1 mM DTT, 200 μM etoposide, and 30 μg/mL bovine serum albumin) at 37°C for 15 min. In the post-strand passage DNA cleavage reaction, 0.5 mM AMP-PNP (Sigma) was added to the reaction mixture. The cleavage reaction was terminated by adding 1% SDS and 0.2 μg/μL proteinase K (Roche). After incubation at 55°C for 1 h, samples were separated on 1% agarose gels, and DNA bands were detected by staining with GelRed Nucleic Acid Gel Stain.

### Clamping assay and western blotting

HEK293 cells transfected with both FLAG and EGFP-tagged proteins were treated with 7 μM ICRF-193 for 15 min in a CO_2_ incubator. Cells were washed with PBS and then harvested in an extraction buffer containing 50 mM HEPES-NaOH (pH 7.4), 1 mM EDTA, 0.5 M NaCl, 0.1% Nonidet P-40, 1 mM dithiothreitol, and 1× Protease inhibitor cocktail (Roche). Soluble and insoluble fractions were separated by centrifugation (15000 rpm) for 10 min at 4°C. Then, SDS sample buffer containing 50 mM Tris-HCl (pH 6.8), 2% SDS, 1.25% 2-mercaptoethanol, 250 mM sucrose, and 0.0025% bromophenol blue was added to each fraction. The samples were separated by SDS-PAGE and transferred to a polyvinylidene difluoride (PVDF; GE Healthcare) membrane. The membranes were incubated with anti-FLAG tag monoclonal antibody (1:1000) (FLAG M2; Sigma) and then with horseradish peroxidase-conjugated secondary antibody (GE Healthcare), followed by detection with ECL Prime Western Blot Detection Kit (GE Healthcare). ImageQuant LAS 4000 mini (GE Healthcare) was used to detect chemiluminescence. Protein bands were quantified by densitometry using Image J software.

### Fluorescence microscopy

For live imaging, HEK293 cells were cultured in a glass-bottom dish (2 × 10^5^ cells/dish). Twenty-four hours after transfection, cells were observed using an Olympus FV3000 confocal microscope equipped with a 40× objective lens (0.95 NA) (Okayama University of Science). FRAP analysis was performed on an Olympus FV3000 confocal microscope equipped with a stage-top CO_2_ incubator (TOKAI HIT). The FRAP module was used for photo-bleach. Fluorescence recovery was recorded using Olympus FV3000 (oil-immersion objective, 60×, 1.3 NA) every 0.3 sec immediately after photo-bleaching the region of interest. Quantification of the fluorescence intensity of the bleached region, the total area of fluorescence, and the background region was performed using cellSens software (Olympus). EasyFRAP-web [23] was used to normalize the fluorescence intensity.

## Results

### DNA binding activity of the 1201–1320 region of rat topo IIβ contributes to efficient catenane formation *in vitro*

**Fig 1:**
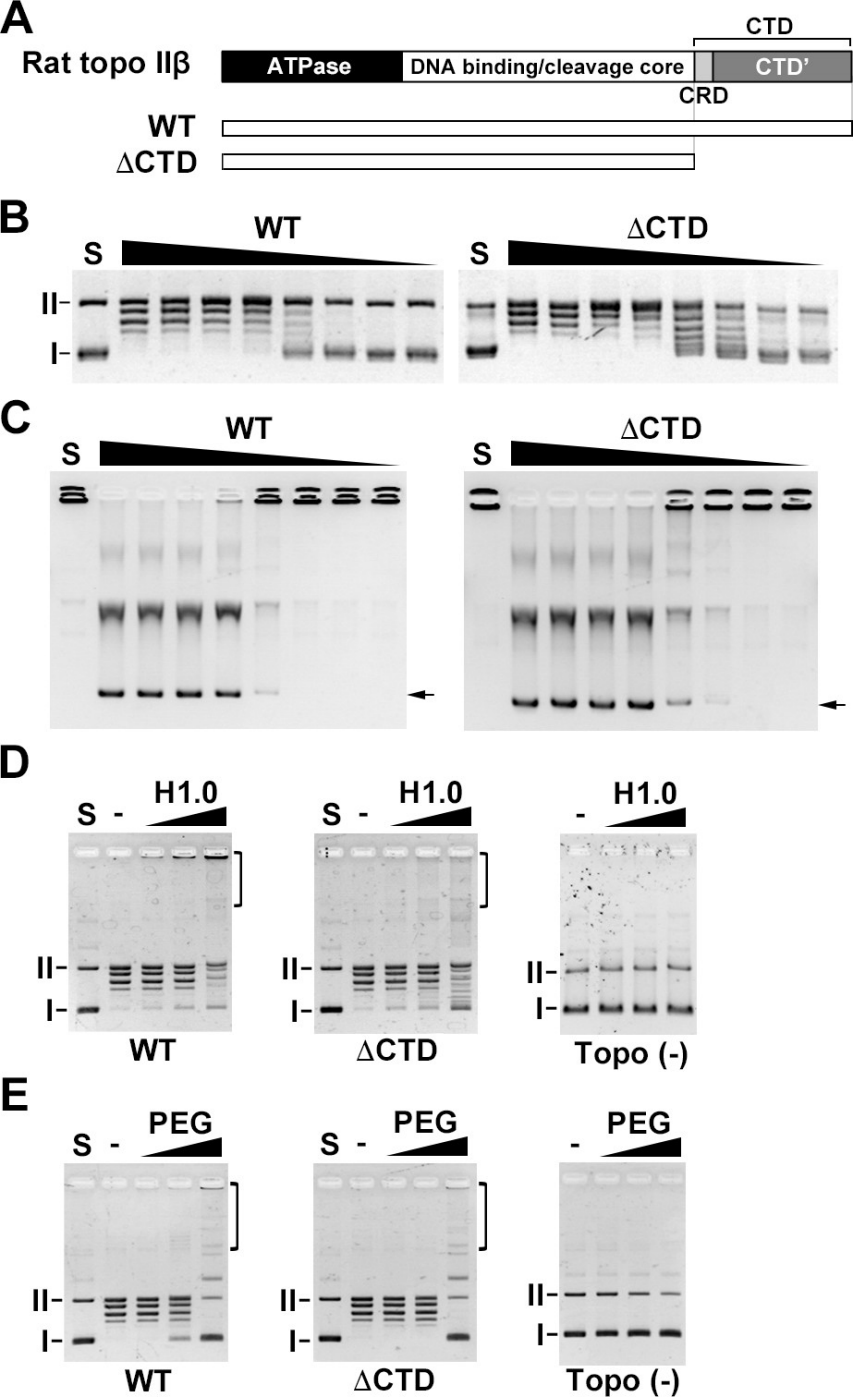
The CTD of rat topo IIβ is required for efficient *in vitro* catenation. (A) Structures of rat topo IIβ and the CTD truncation mutant. (B) The relaxation assay was performed with 2-fold serially-diluted enzyme (2^0^ = 100 fmol) and 50 ng pUC18. (C) The decatenation assay was performed with 2-fold serially-diluted enzyme (2^0^ = 100 fmol) and 100 ng kDNA. (D) Purified FLAG-tagged proteins (100 fmol) were used for catenation assay in the presence of histone H1.0 (1, 2 and 4 μg/mL) as a DNA aggregation factor. Deproteinized samples were analyzed by 1% agarose gel. (E) Catenation assay in the presence of PEG (1, 5 and 10%). I: supercoiled DNA. II: nicked circular DNA. Brackets indicate catenanes.

Topo II catalyzes the catenation of plasmid DNA *in vitro* in the presence of a DNA condensing agent such as histone H1 [24]. Recently, we showed that the DNA binding activity of the CTD of rat topo IIα is required for the efficient formation of DNA catenanes *in vitro* [22]. Therefore, we hypothesized that the DNA binding activity of the CTD of rat topo IIβ could also contribute to the efficient formation of DNA catenanes. To test this idea, we performed catenation assays using FLAG-tagged recombinant topo IIβ WT enzyme (full length rat topo IIβ) and its truncated CTD mutant (ΔCTD) (Fig 1A). First, we performed relaxation and decatenation assays using 2-fold serial dilutions of each enzyme. The relaxation activity of ΔCTD was slightly higher than WT (Fig 1B). This result is consistent with a previous report [21]. The decatenation activity of ΔCTD was also slightly higher than WT (Fig 1C). These results indicate that the truncation of CTD slightly affects the relaxation and decatenation activity of rat topo IIb *in vitro*, but the differences in activity are not significant. Next, we performed catenation assays in the presence of histone H1.0 (H1.0), as described previously [22]. WT enzyme produced slow-migrating, high-molecular-weight DNA (Fig 1D). In contrast, ΔCTD showed only small amounts of slow-migrating, high-molecular-weight DNA (Fig 1D). To examine whether the high-molecular-weight DNA is the catenane of pUC18, the DNA was purified and treated with the restriction endonuclease HindIII, as described previously [Supplemental data in 22]. The HindIII-digested DNA bands corresponded with linearized pUC18 (S1 Fig). This result indicates that the high-molecular-weight DNA is indeed the catenane of pUC18.

In the catenation assay using H1.0 as a condensing agent, the direct interaction between topo IIβ and H1.0 may promote *in vitro* catenation efficiency. Thus, to examine whether rat topo IIβ directly interacts with H1.0, we performed pull-down assays using GST-tagged H1.0. The result of the pull-down assay indicated that topo IIβ did not directly bind to H1.0 (S2 Fig). Therefore, the DNA binding activity of rat topo IIβ via the CTD domain affected the formation of catenanes.

We showed that increasing the local enzyme concentration at DNA is a key to produce catenane *in vitro* using the truncated CTD mutant of rat topo IIα [22]. Since the ΔCTD mutant of rat topo IIβ has relaxation and decatenation activity comparable to WT activity (Fig 1B and 1C), ΔCTD can also produce DNA catenanes by increasing the local enzyme concentration at the DNA. To examine whether the ΔCTD can produce DNA catenane, we used polyethylene glycol (PEG), as described previously [22]. PEG has a macromolecular crowding effect [25], which increases the local enzyme concentration at DNA. Both WT and ΔCTD enzymes produced similar slow-migrating DNA bands and high-molecular-weight DNA (Fig 1E). Therefore, the DNA binding activity of the rat topo IIβ CTD concentrates the enzyme in the vicinity of condensed DNA, enabling the efficient formation of DNA catenanes *in vitro*.

**Fig 2:**
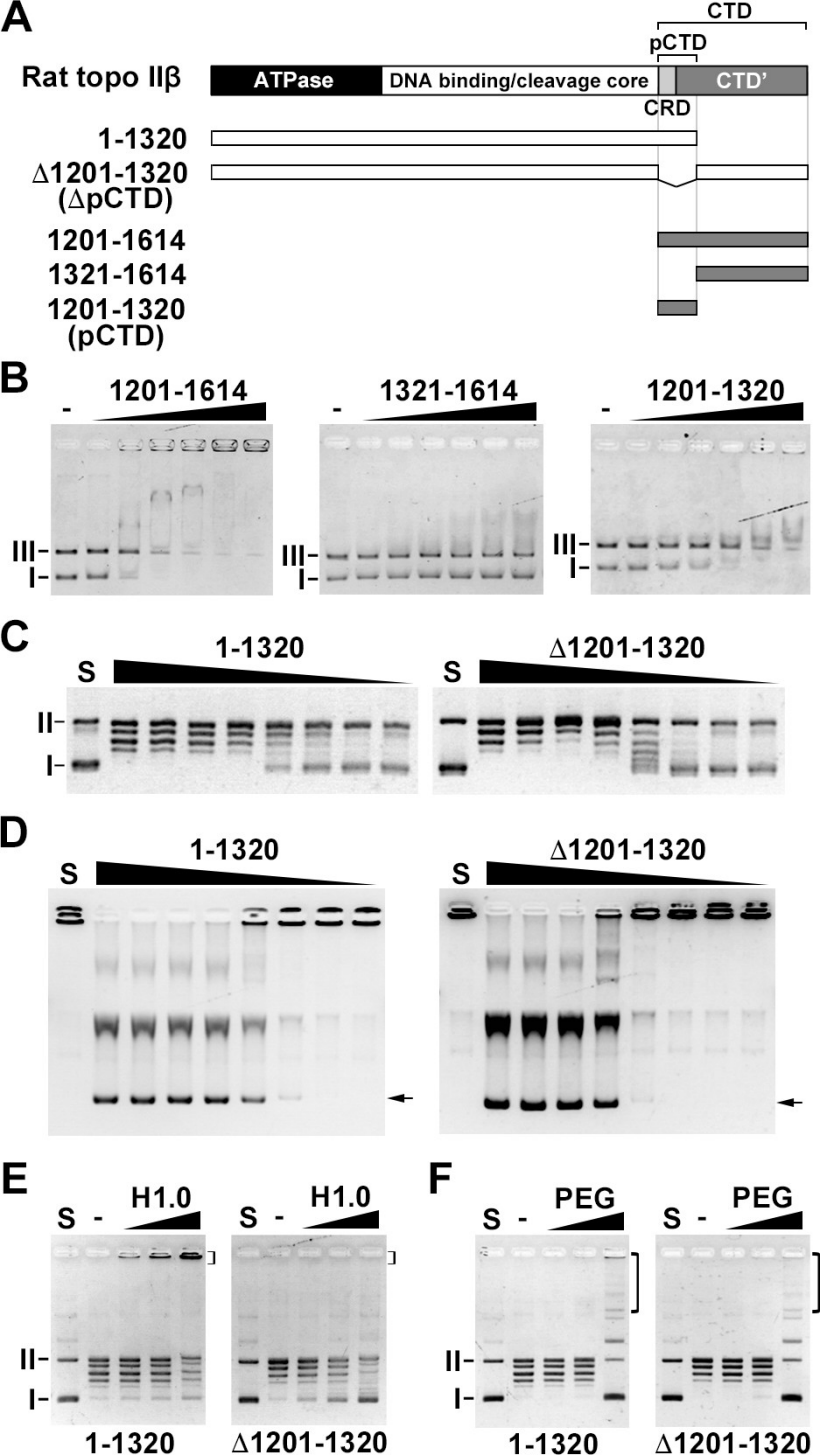
The DNA binding activity of the rat topo IIβ 1201–1320 region (pCTD) contributes to efficient *in vitro* catenation. (A) Scheme of rat topo IIβ CTD (1201–1614) and its truncation mutants used in this study. (B) GST-tagged proteins (100, 200, 300, 400, 500, and 600 fmol) were mixed with 5 ng supercoiled and linearized pUC18 and incubated for 30 min. The reaction mixture was subjected to 1% agarose gel containing MgCl_2_. (C) The relaxation assay was performed as described in Fig 1. (D) The decatenation assay was performed as described in Fig 1. Arrows indicated decatenated DNA bands. (E) The catenation assay was performed in the presence of H1.0 (1, 2, and 4 μg/mL) as described in Fig 1. (F) Catenation assay in the presence of PEG (1%, 5%, and 10%) as described in Fig 1. I: supercoiled DNA. II: nicked circular DNA. III: linearized DNA. Brackets indicate catenanes.

To elucidate which region in the CTD of rat topo IIβ is required for the formation of catenanes, we used ΔCTD’ (1–1250) and ΔCRD (Δ1201–1250) mutants. Since the ΔCTD’ and ΔCRD have DNA binding activity [21], both mutants formed catenanes (S3 Fig). Andrew *et al*. showed that the 1359–1621 region of human topo IIβ CTD has little DNA-binding activity [13]. Therefore, we speculated that the DNA-binding activity would be enclosed in the N-terminus of the CTD’ (1251–1614). At the N-terminal of the CTD' (1251–1320), there are clusters of positively charged amino acids (lysine and arginine) (S4 Fig). Therefore, it was considered that this region might contribute to the binding with negatively charged DNA. To examine whether the 1201–1320 region has DNA-binding activity, we performed an EMSA using GST-tagged recombinant proteins (Fig 2A). The rat topo IIβ CTD domain (1201–1614 and 1201–1320) showed DNA band shifts in a dose-dependent manner (Fig 2B). The 1231–1614 fragments showed a smaller DNA band shift. This result indicates that the 1201–1320 region has DNA binding activity. To test whether the DNA-binding activity of the 1201–1320 region contributes to *in vitro* catenation, we performed the catenation assay using the 1–1320 and the Δ1201–1320 constructs. Before carrying out the catenation assay, we examined the relaxation and decatenation activities of both mutants (Fig 2C and 2D). The relaxation and decatenation activities of the 1–1320 and the Δ1201–1320 mutants were comparable to WT activity. While the 1–1320 mutant formed catenanes in the presence of H1.0, the Δ1201–1320 mutant did not (Fig 2E). In the presence of PEG, both the 1–1320 and the Δ1201–1320 mutants produced catenanes (Fig 2F). Taken together, these data suggest that the CTD of rat topo IIβ, and specifically the 1201–1320 region, contributes to the enzyme binding to condensed DNA, which allows the formation of catenanes. In subsequent experiments, we refer to the 1201–1320 region of rat topo IIβ as the proximal CTD (pCTD).

### The DNA binding activity of pCTD assists in establishing pre-strand passage DNA cleavage

**Fig 3:**
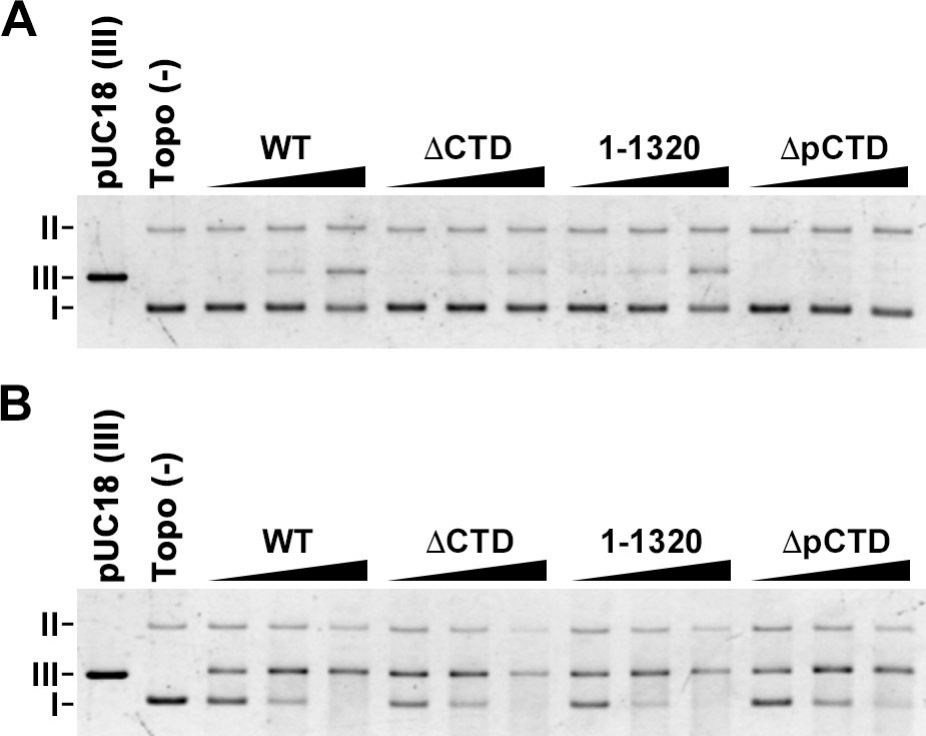
The pCTD of rat topo IIβ supports pre-strand passage DNA cleavage activity. (A) DNA cleavage assay was performed in the absence of ATP. FLAG-tagged protein (50, 100, and 200 fmol) was mixed with 5 ng pUC18 in the presence of 200 μM etoposide and incubated at 37°C for 15 min. Deproteinized samples were analyzed by 1% agarose gel. (B) DNA cleavage assays were performed in the presence of AMP-PNP as described in (A). I: supercoiled DNA. II: nicked circular DNA. III: linearized DNA.

The CTD of human topo IIα affects DNA cleavage activity *in vitro*. The DNA cleavage activity of the truncated CTD human topo IIα enzyme is higher than that of the full-length enzyme [14]. In human topo IIβ (S165R mutant), the drug cleavage sites (e.g., mitoxantrone) of the enzyme are affected by the truncation of its CTD (1263–1621) [26]. These studies suggest that the rat topo IIβ CTD may affect its DNA cleavage reaction *in vitro*. Thus, we examined whether the CTD of rat topo IIβ affects DNA cleavage activity using WT enzyme and its CTD truncation mutants. Eukaryotic topo II establishes two distinct DNA cleavage/replication equilibria: a pre-strand passage DNA cleavage and a post-strand passage DNA cleavage [27]. DNA cleavage assays carried out in the absence of ATP evaluate pre-strand passage DNA cleavage, and assays performed in the presence of a nonhydrolyzable ATP analog, AMP-PNP, evaluates post-strand passage DNA cleavage. Since the percentage of DNA cleavage is usually very low, we used etoposide, which stabilizes a cleavable complex, to enhance DNA cleavage. In the absence of ATP, the WT enzyme produced linearized DNA bands in a dose-dependent manner (Fig 3A). The ΔCTD mutant showed lower DNA cleavage activity than the WT enzyme. While the 1–1320 showed DNA cleavage activity similar to WT, the ΔpCTD showed less DNA cleavage activity than ΔCTD, suggesting that the 1321–1614 domain might suppress pre-strand passage DNA cleavage. The data suggest that the pCTD of rat topo IIβ is involved in establishing the pre-strand DNA cleavage/re-ligation equilibrium. In contrast, in the presence of AMP-PNP, the WT and all mutants showed similar DNA cleavage activity (Fig 3B). Therefore, the rat topo IIβ CTD did not affect the post-strand passage DNA cleavage/re-ligation equilibrium.

In the on-bead DNA binding assay, the ΔCTD bound supercoiled DNA similar to WT (S5A Fig), consistent with a previous study [21]. In EMSA, whereas the WT showed a dose-dependent DNA band shift, ΔCTD showed a smaller DNA band shift (S5B Fig). EMSA is known to detect stable protein–DNA complexes during gel electrophoretic fractionation [28]. Therefore, we considered that the CTD of rat topo IIβ may contribute to the formation of a stable complex between the enzyme and the DNA. To test whether the DNA band shift observed in WT would be observed in ΔCTD if a stable protein–DNA complex is formed, we used AMP-PNP to form a stable closed-clamp intermediate. In the presence of AMP-PNP, ΔCTD showed a DNA band shift comparable to that of WT (S5C and S5D Fig), indicating that ΔCTD is able to bind supercoiled DNA but might not be able to form a stable protein–DNA complex like WT. Moreover, the same results were observed in 1–1320 and ΔpCTD (S5D Fig). These data suggest that the CTD (pCTD) of rat topo IIβ may contribute to form a stable complex between the enzyme and DNA and allow the establishment of the DNA cleavage/re-ligation equilibrium.

**Fig 4:**
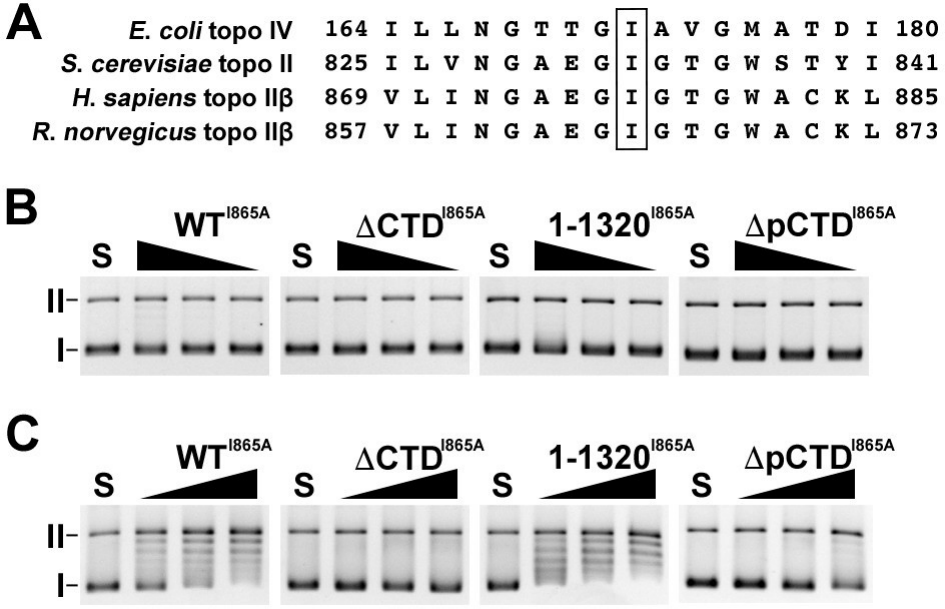
The pCTD of rat topo IIβ maintains the relaxation activity of I865A mutants. (A) Amino acid sequence alignment of *E*. *coli* topo IV ParC subunit (164–180), S. cerevisiae topo II (825–841), H. sapiens topo IIβ (869–885) and R. norvegicus topo IIβ (857–873). Box represents the conserved isoleucine (I). (B) Relaxation assays were performed using 50 ng pUC18 and purified FLAG-tagged I865A mutants (100, 50, and 25 fmol). The DNA bands were detected by GelRed staining. (C) Relaxation assays were performed using 5 ng pUC18 and purified FLAG-tagged I865A mutants (50, 100, and 200 fmol). The DNA bands were detected by SYBR Green I. I: supercoiled DNA. II: nicked circular DNA.

It is known that topo II bends the captured G-segment during the DNA cleavage process from the crystal structure analysis [29–31], and DNA cleavage by topo II is regulated through tight coordination with DNA bending [32]. The DNA-bending step is present between the DNA binding step and the pre-strand passage DNA cleavage/re-ligation equilibrium step of the topo II catalytic cycle. Therefore, the DNA binding activity of CTD and pCTD of rat topo IIβ may also influence the DNA bending step of the enzyme. I172 in the ParC domain of *E*. *coli* topo IV is important for its DNA-bending process [33]. The I172A mutant of *E*. *coli* topo IV reduces DNA-bending activity, and shows low DNA cleavage and supercoiled DNA relaxation activities. We speculated that the relaxation reaction of a mutant with reduced DNA bending activity may be maintained if the DNA binding activity of the pCTD stabilizes the complex during the DNA bending step. To test this idea, we used an I865A mutant of rat topo IIβ, which corresponds to I172A of *E*. *coli* topo IV (Fig 4A). We first carried out the relaxation assay using 2-fold serially-diluted enzyme (2^0^ = 100 fmol). While only a few relaxed bands were observed in the first lane of WT^I865A^ and 1-1320^I865A^ (100 fmol), relaxed bands were not observed in the ΔCTD^I865A^ and ΔpCTD^I865A^ mutants (Fig 4B). This result suggests that the DNA-bending activity was reduced by an I865 mutation in rat topo IIβ. In I172A mutant of *E*. *coli* topo VI, relaxed DNA was slightly observed by increasing the molar ratio of enzyme to DNA [33], so we then changed the molar ratio between enzyme and substrate, and performed the relaxation assay again. With WT^I865A^ and 1-1320^I865A^, relaxed bands were detected in an enzyme concentration-dependent manner (from left to right), while with ΔCTD^I865A^ and ΔpCTD^I865A^ only a few relaxed bands were detected (Fig 4C). These results indicate that the CTD, particularly the pCTD of rat topo IIβ, maintains the relaxation activity of the I865A mutant.

### The pCTD deletion mutant has altered dynamics in the nuclei

**Fig 5:**
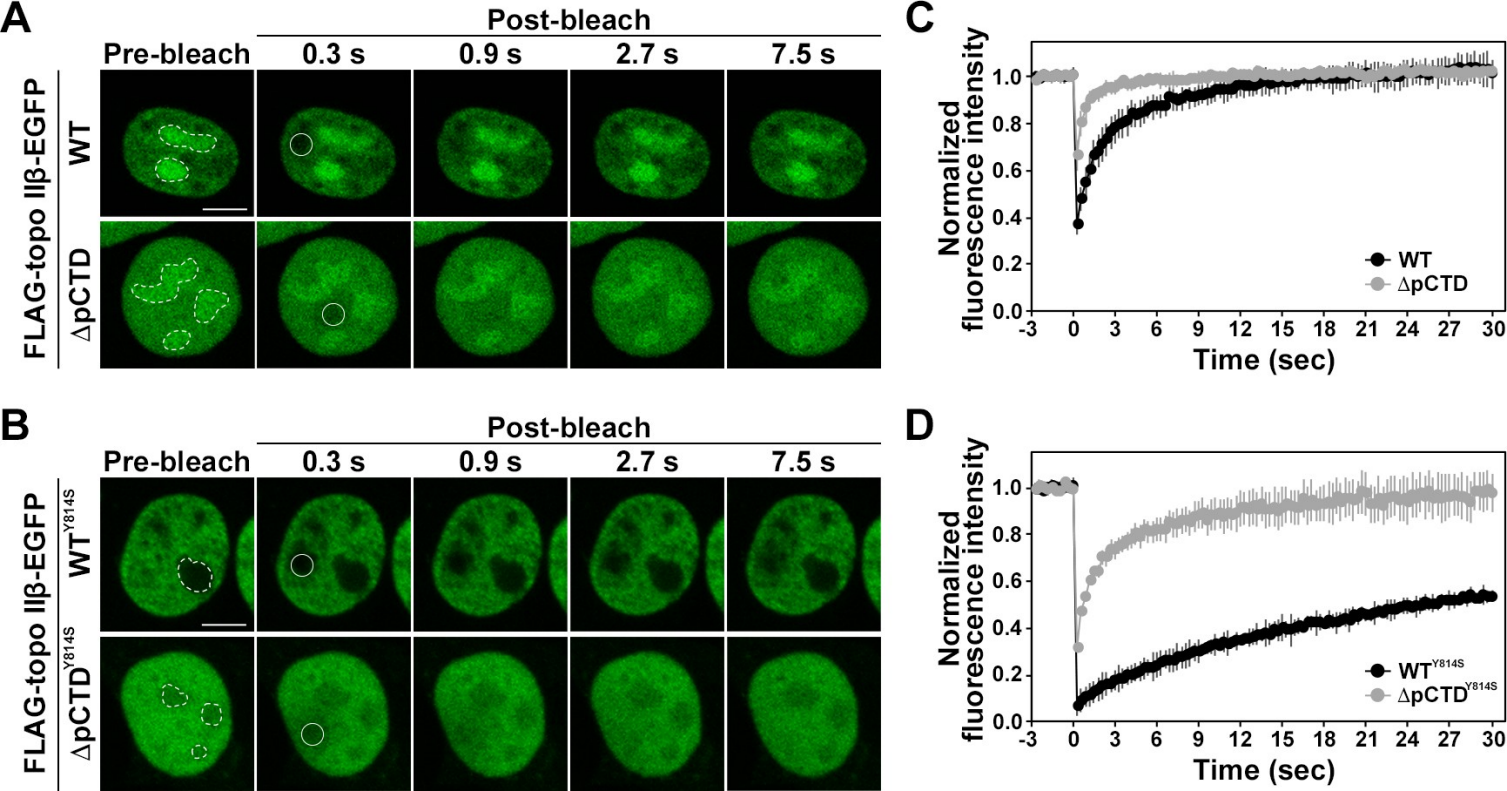
FRAP analysis of FLAG-topo IIβ-EGFP. (A) Fluorescence recovery kinetics of WT (n = 9) and ΔpCTD (n = 12) in HEK293 cells. The photobleached region is indicated by a circle. Scale bars = 5 μm. Dotted lines denote nucleoli. (B) Fluorescence recovery kinetics of WT^Y814S^ (n = 5) and ΔpCTR^Y814S^ (n = 6) in HEK293 cells. Dotted lines denote nucleoli. (C, D) Normalized fluorescence intensities are plotted versus time (s). Values represent mean ± S.D.

In interphase cells, topo IIβ localizes to nucleolus and nucleoplasm and shuttles between nucleolus and nucleoplasm [34]. We first compared the nuclear localization patterns of FLAG-topo IIβ-EGFP (WT) and FALG-topo IIβ ΔpCTD-EGFP (ΔpCTD). ΔpCTD localized to the nucleoplasm and nucleolus, as in WT, but the contrast between nucleoplasm and nucleolus was smaller than in WT (Fig 5A, pre-bleach panel). This phenomenon was thought to be because ΔpCTD lacked the CRD required for localizing to nucleolus [21].

The pCTD of rat topo IIβ is involved in DNA binding *in vitro*. Topo IIβ shuttles between the nucleoplasm and the nucleolus in interphase cells, and associates with DNA in nucleoplasm [21]. Thus, we speculated that the truncation of pCTD may affect the mobility of rat topo IIβ in the nucleoplasm. To determine whether the pCTD affects the mobility of rat topo IIβ, we performed FRAP analysis. As shown in Fig 5A and 5C, the recovery of the fluorescent signal of WT in the nucleoplasm was observed within a few seconds (t_1/2_ = 2.1 ± 0.7 sec), consistent with previous studies [21, 34]. The fluorescent signal of ΔpCTD recovered in a shorter time (t_1/2_ = 0.5 ± 0.1 sec) than that of WT (Fig 5B and 5D), suggesting that the pCTD of topo IIβ is involved in the interaction with nucleoplasmic structures (likely DNA).

The results of on-bead DNA binding assays and EMSA (S5 Fig) suggest that the CTD of rat topo IIβ might contribute to stable DNA binding. Thus, increasing the mobility of ΔpCTD may be caused by a lack of stable DNA binding. To examine whether the pCTD of rat topo IIβ contributes to stable DNA binding in the nucleoplasm, we used a catalytic core mutant of rat topo IIβ (Y814S mutant). The Y814S mutant cannot cleave DNA but can close the N-gate, thus retaining the enzyme in the nucleoplasm by capturing DNA [21, 35]. If the pCTD of rat topo IIβ is involved in stable DNA binding, the fluorescent recovery time of ΔpCTD^Y814S^ may be shorter than WT^Y814S^ after photo-bleaching in FRAP analysis. WT^Y814S^ localized to the nucleoplasm, as previously reported [21]. Notably, fluorescent signal recovery of WT^Y814S^ was significantly slower than that of WT, suggesting that WT^Y814S^ was trapped on DNA, and its mobility was restricted (Fig 5B and 5D). Unlike WT^Y814S^, ΔpCTD^Y814S^ localized to the nucleoplasm and nucleolus (Fig 5B, pre-bleach panel). ΔpCTD^Y814S^ showed more rapid recovery of the fluorescent signal than that of WT^Y814S^ (Fig 5B and 5D). However, the recovery of the fluorescence intensity of ΔpCTD^Y814S^ was slower than that of ΔpCTD, suggesting that a part of ΔpCTD^Y814S^ was trapped on DNA by closing the N-gate. The higher nucleoplasmic mobility of ΔpCTD^Y814S^ than that of WT^Y814S^ suggests that ΔpCTD^Y814S^ hardly binds to DNA or dissociates from DNA before it is trapped on the DNA by closing the N-gate.

### The pCTD affects the formation of the closed-clamp intermediate caused by ICRF-193 treatment

**Fig 6:**
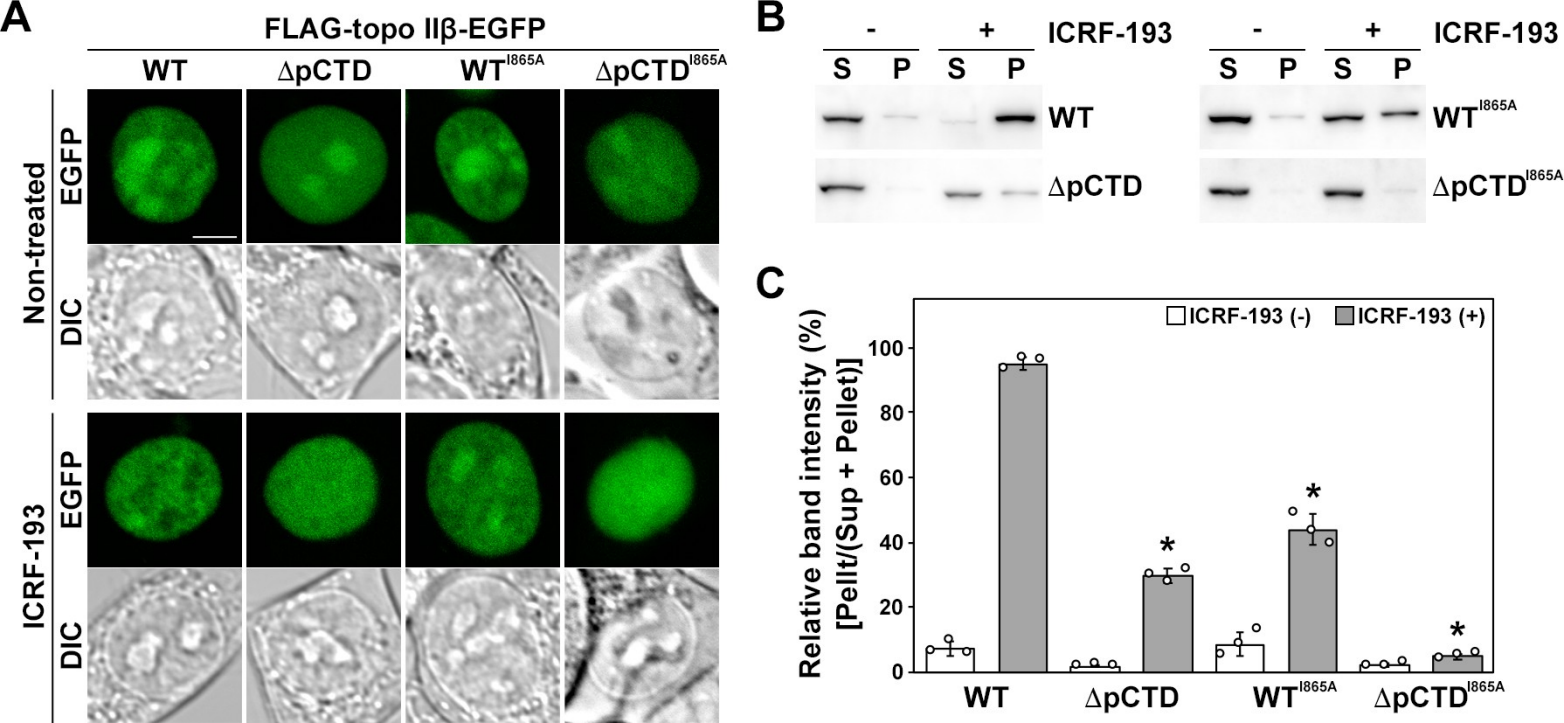
Formation of salt-stable closed-clamp intermediates induced by ICRF-193 treatment. (A) HEK293 cells in a 35-mm glass-bottom dish were transfected with the FLAG-topo IIβ-EGFP expression vector. The live cells were imaged using a 40× objective lens. ICRF-193 (7 μM) was added for 15 min. Scale bar = 5 μm. (B) The clamping assay was performed as described in materials and methods. FLAG and EGFP-tagged proteins were detected by using the anti-FLAG tag antibody. (C) Relative band intensities in (B) were calculated from band densitometry using Image J software. The experiments were performed in triplicate, and the results are indicated as mean ± S.D. Circles represent individual data points. Asterisks (P < 0.001) indicate a significant deviation from WT (Student’s t-test).

ICRF-193 is a catalytic inhibitor of topo II that blocks ATP hydrolysis, thereby inducing the formation of a closed-clamp intermediate of topo II on DNA [36]. Treatment with ICRF-193 also induces subnuclear relocation of topo IIβ from the nucleolus to the nucleoplasm and restricts its nuclear mobility [21, 37]. Upon treatment with ICRF-193, the fluorescent signal of WT FLAG-topo IIβ-EGFP relocated from the nucleolus to the nucleoplasm (Fig 6A). However, in the same conditions, the fluorescent signal of ΔpCTD did not show a relocation as clear as that of WT (Fig 6A). This suggests that the pCTD of rat topo IIβ affected the formation of the closed-clamp intermediate induced by ICRF-193 treatment. The closed-clamp intermediates induced by ICRF-193 treatment are known to be salt-stable [36]. Therefore, to clarify whether the pCTD of rat topo IIβ affects the closed-clamp formation caused by ICRF-193 treatment, we performed the clamping assay, a method based on the fact that closed-clamp intermediates generated by ICRF-193 treatment cannot easily be solubilized in a high salt buffer. Without treatment, WT was detected in the soluble fraction (sup), but after ICRF-193 treatment, it was mostly found in the insoluble fraction (pellet) (Fig 6B and 6C). ΔpCTD was solubilized by high salt in the same manner as WT; however, upon ICRF-193 treatment, the relative amount of ΔpCTD fractionated in the pellet was much lower than that of WT (Fig 6B and 6C). These data corroborate the results in Fig 6A, suggesting that the pCTD affects the formation of a closed-clamp intermediate induced by ICRF-193 treatment.

Incompetent topo IIβ, such as the G173I mutant of rat topo IIβ, lacking ATP-binding activity and WT rat topo IIβ in ATP-depleted cells, do not undergo subnuclear relocation induced by ICRF-193 treatment [21]. Accordingly, WT^I865A^, which reduces DNA-bending activity, remained in the nucleolus to some extent after ICRF-193 treatment (Fig 6A). Moreover, the closed-clamp intermediate formed with this mutant was only about half of that observed with WT (Fig 6B and 6C), suggesting that the DNA-bending activity of topo IIβ is required to form the closed-clamp intermediate induced by ICRF-193. ΔpCTD^I865A^ showed the same localization pattern as ΔpCTD in both non-treated and ICRF-193-treated cells (Fig 6A). Furthermore, ΔpCTD^I865A^ was hardly detected in the pellet after ICRF-193 treatment (Fig 6B and 6C). Since ΔpCTD^I865A^ lacks DNA binding and reduces DNA bending activity, this mutant does not form closed-clamp intermediates upon ICRF-193 treatment. Collectively, these results suggest that the binding of rat topo IIβ to DNA via pCTD is required for the enzyme to enter the catalytic cycle, which consequently affects the formation of closed-clamp intermediates caused by ICRF-193 treatment.

## Discussion

In this study, we found that the pCTD of rat topo IIβ has DNA binding activity that allows efficient *in vitro* catenation. Recently, we have shown that the CTD of rat topo IIα, which contains several clusters of positively charged amino acids, is involved in DNA binding and in the efficient formation of catenane *in vitro* [22]. Similarly, clusters of positively-charged amino acids are present in the CTD of rat topo IIβ. The presence of multiple nuclear localization signals in the CTD of human topo IIβ has been shown by using a β-galactosidase reporter system [38]. In the human topo IIβ CTD, two strong NLSs are found at 1522–1548 and 1538–1573, and a weak sequence at 1294–1332 (S4 Fig). The rat topo IIβ pCTD we defined in this study contains the 1287–1325 region corresponding to the 1294–1322 region in human topo IIβ, showing weak NLS activity. Besides, pCTD also includes CRD with lysine-cluster at each end. Therefore, positively charged amino acids present in the rat topo IIβ pCTD may contribute to DNA-binding, thereby enabling efficient *in vitro* catenation. Additionally, ΔpCTD did not produce DNA catenanes in the presence of H1.0, despite having two strong NLSs, suggesting that the position of the positively-charged amino acid clusters in the CTD might be also important.

We also found that the pCTD contributes to establishing the pre-strand passage DNA cleavage complex *in vitro* (Fig 3). The on-bead DNA binding and the EMSA data using ΔCTD in S5 Fig suggest that the CTD of rat topo IIβ helps form the stable complex between topo IIβ and DNA. Thus, the truncation mutant of CTD (or pCTD) may dissociate from DNA before establishing pre-strand passage DNA cleavage. It has been suggested that DNA-bending by topo II allows a precise DNA cleavage reaction linked to the subsequent closing of the N-gate [32, 33]. The fact that the efficiency of closed-clamp formation of WT^I865A^ induced by ICRF-193 treatment was approximately half than that of WT (Fig 6), suggesting that the reduced DNA-bending activity of topo IIβ makes it difficult to proceed to the N-gate closing step in nuclei. As expected, ΔpCTD^I865A^ failed to form the closed-clamp intermediate. These results are consistent with the ‘DNA-binding-bending-cleavage’ model [32], and further suggest that the pCTD may be involved in the progression of the catalytic reaction of topo IIβ by stabilizing the complex formed between the enzyme and DNA. While the catalytic activity of topo II is essential for maintaining the integrity of the genome, transient double-stranded DNA cleavage by the enzyme can potentially cause cell death and chromosomal translocation [39]. Thus, the DNA-cleaving activity of topo II in nuclei needs to be tightly controlled.

FRAP data of the ΔpCTD indicated higher nucleoplasmic mobility than that of WT (Fig 5). Interactions between topo IIβ and cellular components have been suggested to be ionic in nature [21]. In the salt extraction experiments with whole cells, WT was efficiently extracted with a dependence on NaCl concentration (S6 Fig). However, ΔpCTD was easily extracted at low concentrations of NaCl, suggesting that the pCTD of topo IIβ contributes to the interaction between the enzyme and the cellular components (presumably, DNA). Therefore, we speculate that the higher nucleoplasmic mobility of ΔpCTD may be due to the lack of interactions with nuclear components. It has been shown that the localization pattern of mCherry-tagged topo IIα ΔChT mutant to mitotic chromosome is different between live cells and fixed cells, suggesting that lack of ChT domain destabilizes the stable binding of topo IIα to mitotic chromosome [13]. Similar data were obtained by fixing the cells expressing EGFP-tagged topo IIβ (WT and ΔpCTD) with paraformaldehyde. The localization pattern of the WT enzyme in the interphase nucleus was not different between live cells (Fig 6) and fixed cells (S7 Fig). However, the localization pattern of ΔpCTD was significantly changed by fixing the cells. The fluorescence signal of ΔpCTD disappeared from the nucleoli and was excluded from the DAPI-stained region in the nucleoplasm. These data suggest that the lack of the pCTD, including the CRD, destabilizes topo IIβ binding to both nucleolar RNA and chromatin DNA, leading to a different localization pattern between live cells and fixed cells. Therefore, the DNA binding of the pCTD of rat topo IIβ may affect stable binding to chromatin DNA in nucleoplasm.

Our data suggest that the CTD of topo IIβ is involved in the stable binding of the enzyme to chromatin DNA. However, in the nucleoplasm, it is unclear how topo IIβ interacts with chromatin DNA through the CTD, directly, or indirectly with other chromatin components. Recently, genome-wide ChIP-seq analysis indicated that histone tail post-translational modifications that are characteristic of active chromatin (e.g., H3K4 methylation) are enriched in the promoter regions of the genes targeted by topo IIβ [40, 41]. Topo IIα interacts with mitotic chromosomes by recognizing histone post-translational modifications through the ChT domain [13]. At the end of the CTD of topo IIβ, there is a region that is partially homologous to the amino acid sequence of topo IIα ChT. Possibly, after recognizing a histone modification via the end of the CTD, topo IIβ forms a stable complex with chromatin DNA through the pCTD.

## Conclusions

*In vitro* catenation assays and EMSA showed that rat topo IIβ pCTD has DNA binding activity. The DNA binding activity contributes to establish the pre-strand passage DNA cleavage/re-ligation equilibrium *in vitro*. FRAP analysis and clamping assays showed that topo IIβ pCTD is required to form a closed-clamp intermediate of the enzyme. Our data indicate that the pCTD-mediated DNA binding of topo IIβ contributes to the catalytic activity of the enzyme in the nucleoplasm.

## Supporting information

S1 Table List of primers used in this study. (PDF)

S1 Fig Digestion of purified DNA catenanes by restriction endonuclease. Catenanes produced by WT FLAG-tagged protein were purified by centrifugation as described previously [1]. Purified catenanes and pUC18 were digested with the restriction endonuclease HindIII (New England Biolabs). Samples were separated on 1% agarose gels. DNA bands were detected by staining with GelRed Nucleic Acid Gel Stain (Biotium). Catenanes were produced in the presence of H1.0 (A) and PEG (B). Brackets indicate catenane. I: supercoiled pUC18. III: linearized pUC18.
(TIF)

S2 Fig GST pull-down assay using GST tagged histone H1.0. GST pull-down assay was performed as previously described [1]. FLAG-tagged topo IIβ WT (100 ng) and GST-histone H1.0 immobilized on MagneGST Glutathione Particles (Promega) were mixed in 30 μL of PD buffer containing 50 mM Tris-HCl (pH 8.0), 120 mM KCl, 10 mM MgCl2, 1 mM DTT, 0.05% NP-40 and protease inhibitor cocktail (Roche). After incubation on ice for 1 hour, the beads were separated from the supernatant using a magnetic stand and washed 5 times with PD buffer. Input, bound, and unbound fractions were subjected to 8.0% SDS polyacrylamide gel. The gel was stained with CBB stain One (Nacalai Tesque). M indicates a lane with molecular weight markers.
(TIF)

S3 Fig ΔCRD and ΔCTD’ form catenane *in vitro*. (A) Scheme of ΔCRD and ΔCTD’. (B) The catenation assay in the presence of histone H1.0 (H1.0) was performed as described in the main text. (C) The catenation assay in the presence of PEG was performed as described in the main text. Brackets indicate catenane. I: supercoiled DNA. II: nicked circular DNA.
(TIF)

S4 Fig Amino acid sequence alignment of human and rat topo IIβ CTD. CRD, NLS1, 2, and 3 are shown in boxes. Lysine (K) and arginine (R) are highlighted in blue.
(PDF)

S5 Fig On-bead DNA binding assay and EMSA. (A) FLAG-tagged proteins (WT and ΔCTD) were immobilized on Dynabeads protein G (Veritas) using FLAG M2 antibody (Sigma), and were mixed with 5 ng pUC18 and incubated at 30°C for 30 min. After incubation, bound DNA was purified from the bead fraction by SDS/PK treatment and phenol/chloroform extraction. The purified DNA was quantified with a Qubit dsDNA HS Kit (Invitrogen). The experiment was performed in triplicate. The results are indicated as mean ± S.D. (B) FLAG-tagged proteins (WT and ΔCTD) were mixed with 5 ng supercoiled pUC18 in 10 μL binding buffer (described in Materials and Methods) and incubated at 30°C for 30 min. After incubation, the reaction mixture was analyzed on 1% agarose gels containing 10 mM MgCl_2_. Tris-borate-EDTA buffer (0.5× concentration) was used for the running buffer. DNA bands were detected by staining with GelRed Nucleic Acid Gel Stain (Biotium). The asterisks indicate protein–DNA complexes. I: supercoiled DNA. (C) EMSA was performed as described in (B) in the presence of 0.5 mM AMP-PNP. In this assay, 200 fmol of protein was used. Brackets indicate protein–DNA complex. (D) EMSA was carried out in the absence (○) or presence (●) of 0.5 mM AMP-PNP. DNA bands were quantitated by band densitometry. The percentage of bound DNA was determined by the ratio of the band density of total DNA minus free DNA versus the total DNA band density.
(TIF)

S6 Fig Salt extraction of topo Iiβ. HEK293 cells were grown in 100 mm dish. After transfection, the cells were harvested in PBS, and then lysed in extraction buffer (50 mM HEPES-NaOH (pH 7.4), 1 mM EDTA, 1 mM dithiothreitol, 0.1% nonidet P-40, and 1× concentration of protease inhibitor cocktail (PIC, EDTA-free; Roche)) containing different concentrations of NaCl (50, 100, 150, 200, and 300 mM). Soluble (S) and insoluble (P) fractions were fractionated by centrifugation. Proteins were detected by using anti-FLAG tag antibody. S: supernatant, P: pellet.
(TIF)

S7 Fig Observation of localization pattern of topo IIβ WT and ΔpCTD in cells fixed with formaldehyde. HEK293 cells were grown on 13-mm coverslips. After transfection, as described in materials and methods in the main text, the cells were fixed with 4% paraformaldehyde at 37°C for 10 min. The coverslips mounted on a slide glass using VECTASHIELD^®^ Antifade Mounting Medium with DAPI (Vector Laboratory). Images were acquired with a 60× oil-immersion objective lens (1.3 NA) on an Olympus FV3000. Scale bar indicates 5 μm.
(TIF)

S1 Raw images (PDF)
