# Supplementary material for: DNA binding activity of the proximal C-terminal domain of rat DNA topoisomerase IIβ is involved in ICRF-193-induced closed-clamp formation
Source: PLoS One. 2020 Sep 22;15(9):e0239466. doi: 10.1371/journal.pone.0239466 (PMC7508362; doi:10.1371/journal.pone.0239466)
Supplement: S1 Table — (PDF) [file pone.0239466.s001.pdf]

S1 Table. List of primers used in this study

primers	Sequence 5’ to 3’
For 1201-1320 fragment	
F-primer	TCCCCCGGGAAAGCAGTGAAAGGCAAA
R-primer 1	TCCCCCGGGTCACCGCTTCTTCACTTTCT
R-primer 2	TCCCCCGGGCCGCTTCTTCACTTTCT
For 1320-1614 fragment	
F-primer	TCCCCCGGGAATCCTTGGTCAGATGA
R-primer 1	GTGCTCCCCGGGCACTTAATTAAACATTGC
R-primer 2	GCTCCCGGGGCACTTCATTAAACATTGC
For 1201-1614 fragment	
F-primer	TCCCCCGGGAAAGCAGTGAAAGGCAAA
R-Primer	GGAATTCTCAATTAAACATTGCAA
For 1201-1320 fragment	
F-primer	TCCCCCGGGAAAGCAGTGAAAGGCAAA
R-primer	GGAATTCTCACCGCTTCTTCACTTTCT
For 1321-1614 fragment	
F-primer	TCCCCCGGGAATCCTTGGTCAGATGA
R-primer	GGAATTCTCAATTAAACATTGCAA
