# Supplementary figures and images for: DNA binding activity of the proximal C-terminal domain of rat DNA topoisomerase IIβ is involved in ICRF-193-induced closed-clamp formation
Source: PLoS One. 2020 Sep 22;15(9):e0239466. doi: 10.1371/journal.pone.0239466 (PMC7508362; doi:10.1371/journal.pone.0239466)

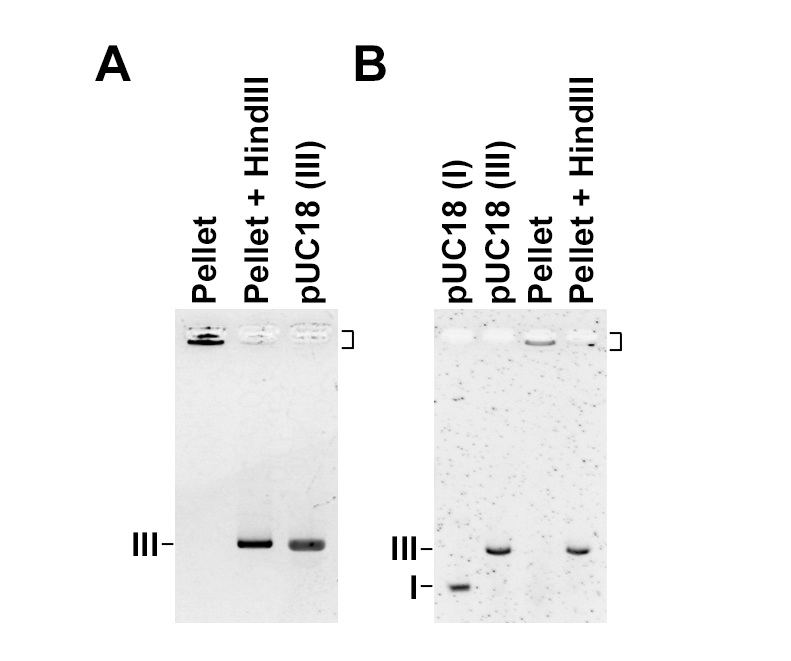

Supplement: S1 Fig — Catenanes produced by WT FLAG-tagged protein were purified by centrifugation as described previously [1]. Purified catenanes and pUC18 were digested with the restriction endonuclease HindIII (New England Biolabs). Samples were separated on 1% agarose gels. DNA bands were detected by staining with GelRed Nucleic Acid Gel Stain (Biotium). Catenanes were produced in the presence of H1.0 (A) and PEG (B). Brackets indicate catenane. I: supercoiled pUC18. III: linearized pUC18. (TIF) [file pone.0239466.s002.tif]

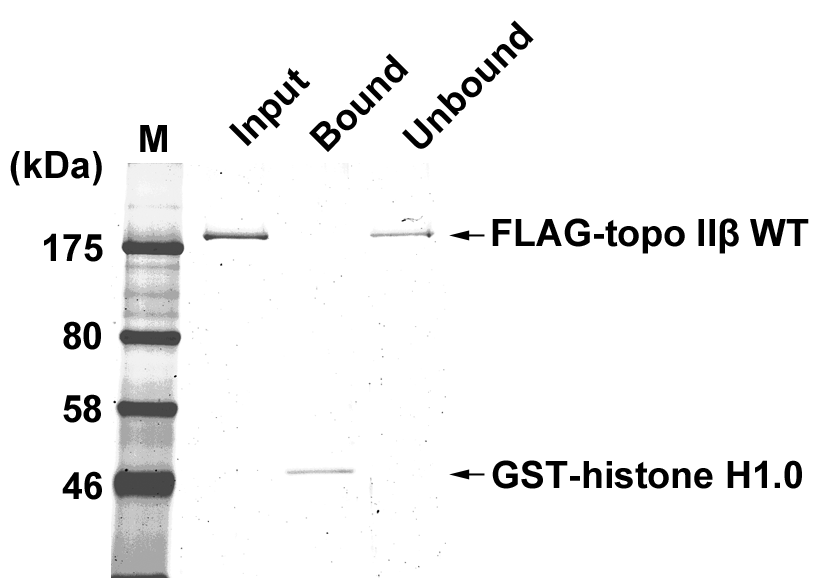

Supplement: S2 Fig — GST pull-down assay was performed as previously described [1]. FLAG-tagged topo IIβ WT (100 ng) and GST-histone H1.0 immobilized on MagneGST Glutathione Particles (Promega) were mixed in 30 μL of PD buffer containing 50 mM Tris-HCl (pH 8.0), 120 mM KCl, 10 mM MgCl2, 1 mM DTT, 0.05% NP-40 and protease inhibitor cocktail (Roche). After incubation on ice for 1 hour, the beads were separated from the supernatant using a magnetic stand and washed 5 times with PD buffer. Input, bound, and unbound fractions were subjected to 8.0% SDS polyacrylamide gel. The gel was stained with CBB stain One (Nacalai Tesque). M indicates a lane with molecular weight markers. (TIF) [file pone.0239466.s003.tif]

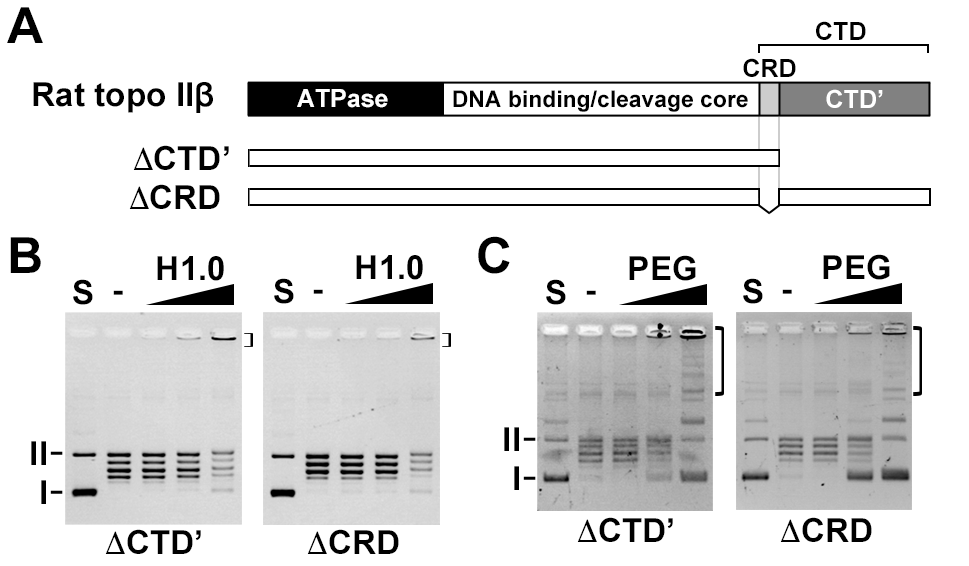

Supplement: S3 Fig — (A) Scheme of ΔCRD and ΔCTD’. (B) The catenation assay in the presence of histone H1.0 (H1.0) was performed as described in the main text. (C) The catenation assay in the presence of PEG was performed as described in the main text. Brackets indicate catenane. I: supercoiled DNA. II: nicked circular DNA. (TIF) [file pone.0239466.s004.tif]

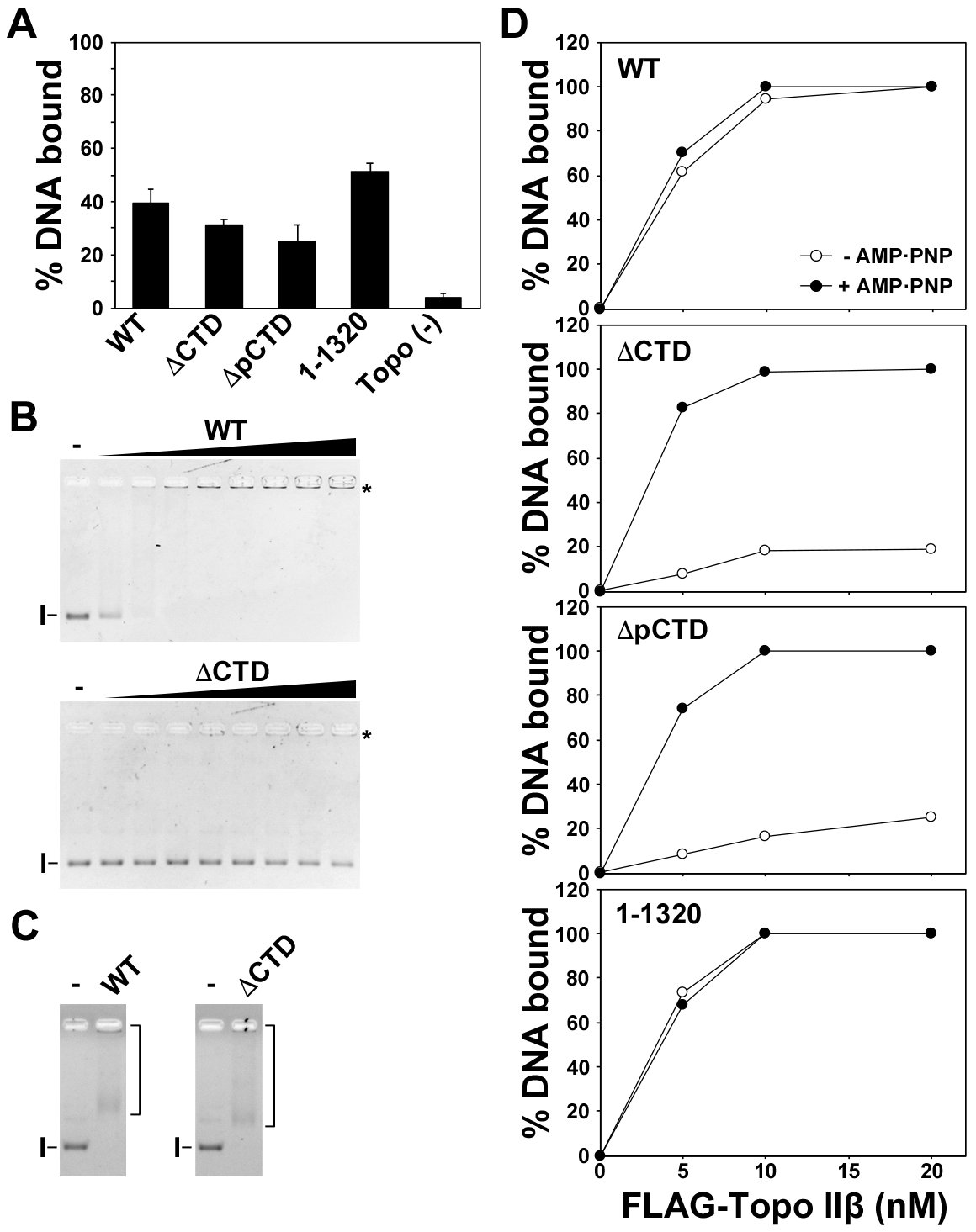

Supplement: S5 Fig — (A) FLAG-tagged proteins (WT and ΔCTD) were immobilized on Dynabeads protein G (Veritas) using FLAG M2 antibody (Sigma), and were mixed with 5 ng pUC18 and incubated at 30°C for 30 min. After incubation, bound DNA was purified from the bead fraction by SDS/PK treatment and phenol/chloroform extraction. The purified DNA was quantified with a Qubit dsDNA HS Kit (Invitrogen). The experiment was performed in triplicate. The results are indicated as mean ± S.D. (B) FLAG-tagged proteins (WT and ΔCTD) were mixed with 5 ng supercoiled pUC18 in 10 μL binding buffer (described in Materials and Methods) and incubated at 30°C for 30 min. After incubation, the reaction mixture was analyzed on 1% agarose gels containing 10 mM MgCl2. Tris-borate-EDTA buffer (0.5× concentration) was used for the running buffer. DNA bands were detected by staining with GelRed Nucleic Acid Gel Stain (Biotium). The asterisks indicate protein–DNA complexes. I: supercoiled DNA. (C) EMSA was performed as described in (B) in the presence of 0.5 mM AMP-PNP. In this assay, 200 fmol of protein was used. Brackets indicate protein–DNA complex. (D) EMSA was carried out in the absence (○) or presence (●) of 0.5 mM AMP-PNP. DNA bands were quantitated by band densitometry. The percentage of bound DNA was determined by the ratio of the band density of total DNA minus free DNA versus the total DNA band density. (TIF) [file pone.0239466.s006.tif]

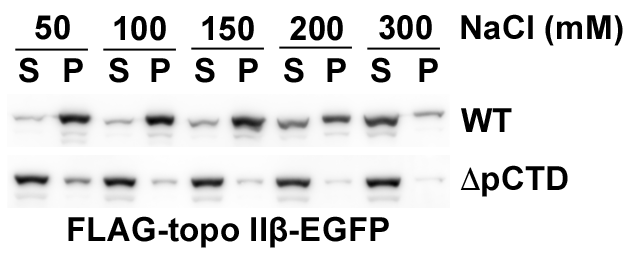

Supplement: S6 Fig — HEK293 cells were grown in 100 mm dish. After transfection, the cells were harvested in PBS, and then lysed in extraction buffer (50 mM HEPES-NaOH (pH 7.4), 1 mM EDTA, 1 mM dithiothreitol, 0.1% nonidet P-40, and 1× concentration of protease inhibitor cocktail (PIC, EDTA-free; Roche)) containing different concentrations of NaCl (50, 100, 150, 200, and 300 mM). Soluble (S) and insoluble (P) fractions were fractionated by centrifugation. Proteins were detected by using anti-FLAG tag antibody. S: supernatant, P: pellet. (TIF) [file pone.0239466.s007.tif]

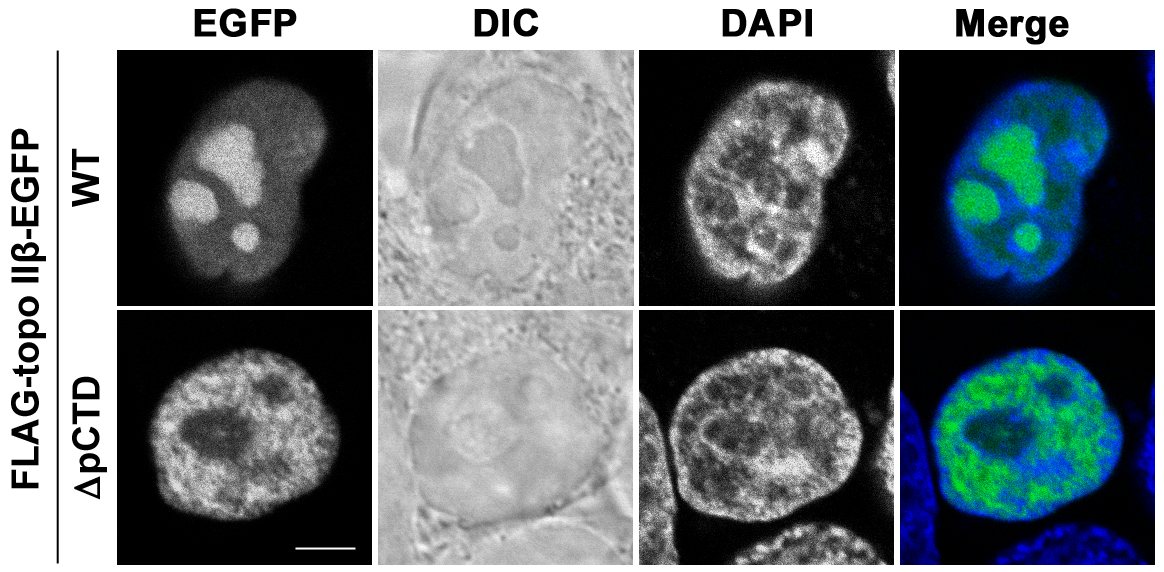

Supplement: S7 Fig — HEK293 cells were grown on 13-mm coverslips. After transfection, as described in materials and methods in the main text, the cells were fixed with 4% paraformaldehyde at 37°C for 10 min. The coverslips mounted on a slide glass using VECTASHIELD® Antifade Mounting Medium with DAPI (Vector Laboratory). Images were acquired with a 60× oil-immersion objective lens (1.3 NA) on an Olympus FV3000. Scale bar indicates 5 μm. (TIF) [file pone.0239466.s008.tif]
