# Supplementary material for: DNA binding activity of the proximal C-terminal domain of rat DNA topoisomerase IIβ is involved in ICRF-193-induced closed-clamp formation
Source: PLoS One. 2020 Sep 22;15(9):e0239466. doi: 10.1371/journal.pone.0239466 (PMC7508362; doi:10.1371/journal.pone.0239466)
Supplement: S4 Fig — CRD, NLS1, 2, and 3 are shown in boxes. Lysine (K) and arginine (R) are highlighted in blue. (PDF) [file pone.0239466.s005.pdf]

```
                  CRD
H. sapiens        SGKAIKGKVGKPKVKKLQLEETMPSPYGRRIIPEITAMKADASKKLLKKKKGDLDTAAVK
R. norvegicus     SGKAVKGKVGKAKVKKLQLEETMPSPYGRRIVPEITAMKADASRKLLKKKKGDPDTTVVK
                   1201                                             1250

                                             NLS1
H. sapiens        VEFDEEFSGAPVEGAGEEALTPSVPINKGPKPKREKKEPGTRVRKTPTSSGKPSAKKVKK
R. norvegicus     VEFDEEFSGTPAEGTGEETLTPSAPVNKGPKPKREKKEPGTRVRKTPASTGKPNTKKVKK

H. sapiens        RNPWSDDESKSESDLEETEPVVIPRDSLLRRAAAERPKYTFDFSEEEDDDADDDDDDNND
R. norvegicus     RNPWSDDESKSESDLEEAEPVVIPRDSLLRRAAAERPKYTFDFSEEEEDDADDDDD-NND
                  1320

H. sapiens        LEELKVKASPITNDGEDEFVPSDGLDKDEYTFSPGKSKATPEKSLHDKKSQDFGNLFSFP
R. norvegicus     LEELKVKASPITNDGEDEFVPSDGIDKDEYAFSPGKSKATPEKSSHDKKSQDFGNLFSFP

H. sapiens        SYSQKSEDDSAKFDSNEEDSASVFSPSFGLKQTDKVPSKTVAAKKGK-PSSDTVPKPKRA
R. norvegicus     SYSQKSEDDSAKFDSNEEDTTSVFAPSFGLKQTDKVPSQTVAAKKGKAPSDAAAPKAKRA

                                  NLS2              NLS3
H. sapiens        PKQKKVVEAVNSDSDSEFG-IPKKTTTPKGKGRGAKKRKASGSENEGDYNPGRKTSKTTS
R. norvegicus     PRQRKVVEPANSDSDSELGNIPKKTAAPKGKGRGAKKRKASGSENEGDYNPGRKPSKTAS

H. sapiens        KKPKKTSFDQDSDVDIFPSDFPTEPPSLPRTGRARKEVKYFAESDEEEDDVDFAMFN
R. norvegicus     KKPKKTSFDQDSDVDIFPSDFTSEPPALPRTGRARKEVKYFAESDEEED-VDFAMFN
```
