# Supplementary material for: DNA binding activity of the proximal C-terminal domain of rat DNA topoisomerase IIβ is involved in ICRF-193-induced closed-clamp formation
Source: PLoS One. 2020 Sep 22;15(9):e0239466. doi: 10.1371/journal.pone.0239466 (PMC7508362; doi:10.1371/journal.pone.0239466)
Supplement: S1 Raw images — (PDF) [file pone.0239466.s009.pdf]

Fig 1B (WT)

MediCap USB170 (MEDI CAPTURE) equipped with CCD camera

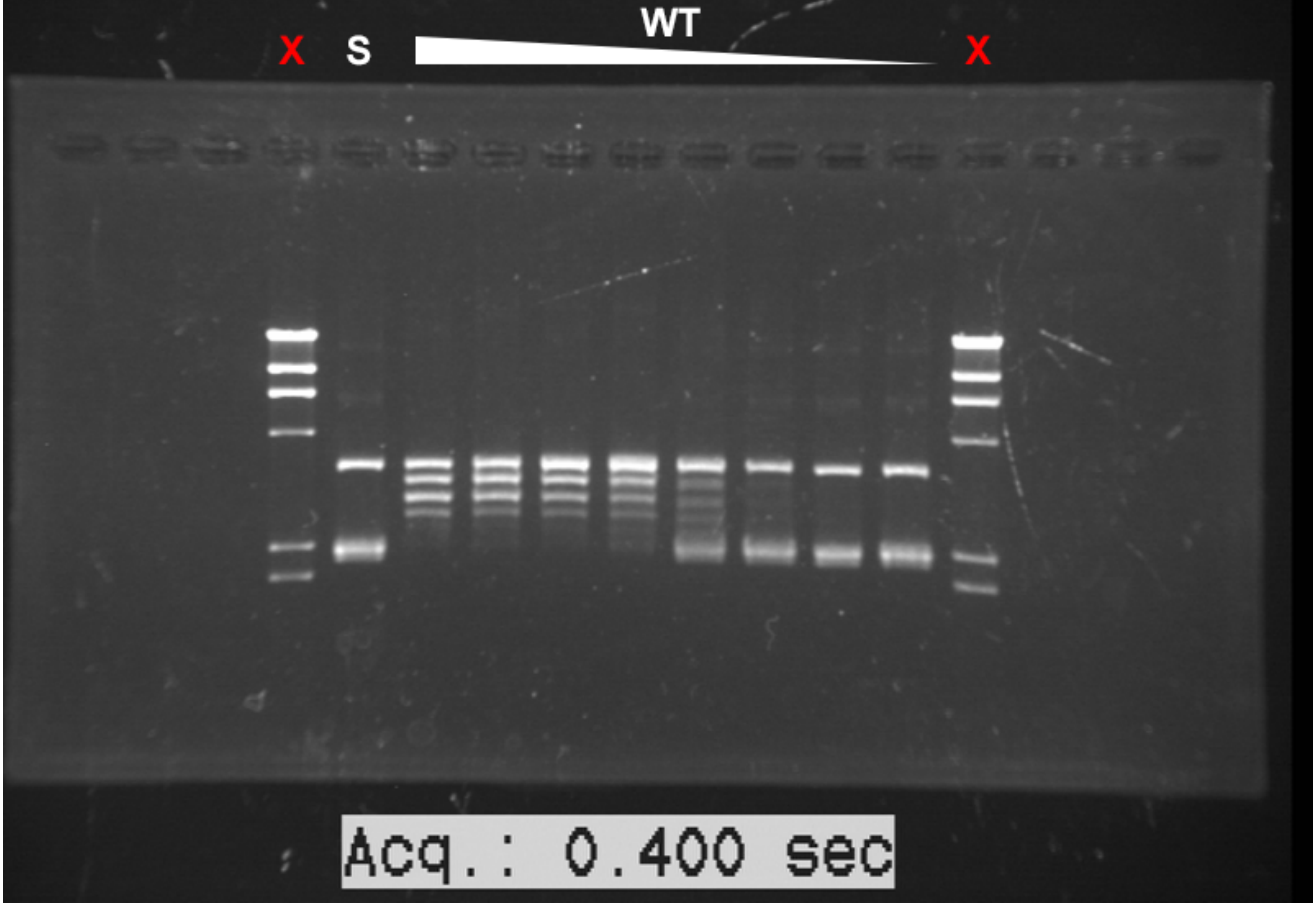

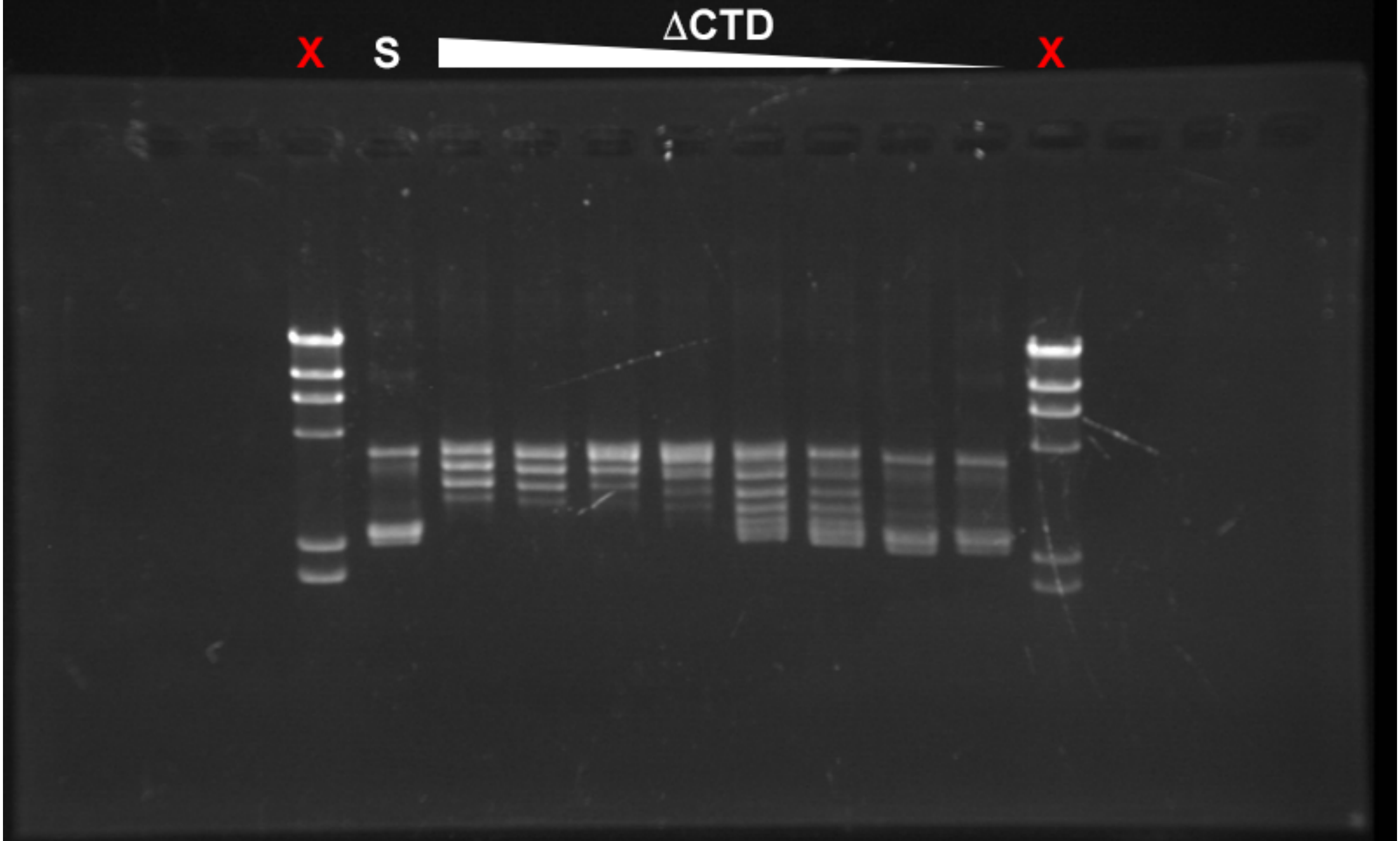

Fig 1B (ΔCTD)

MediCap USB170 (MEDI CAPTURE) equipped with CCD camera

Acq. : 0.400 sec

Fig 1C (WT)

MediCap USB170 (MEDI CAPTURE) equipped with CCD camera

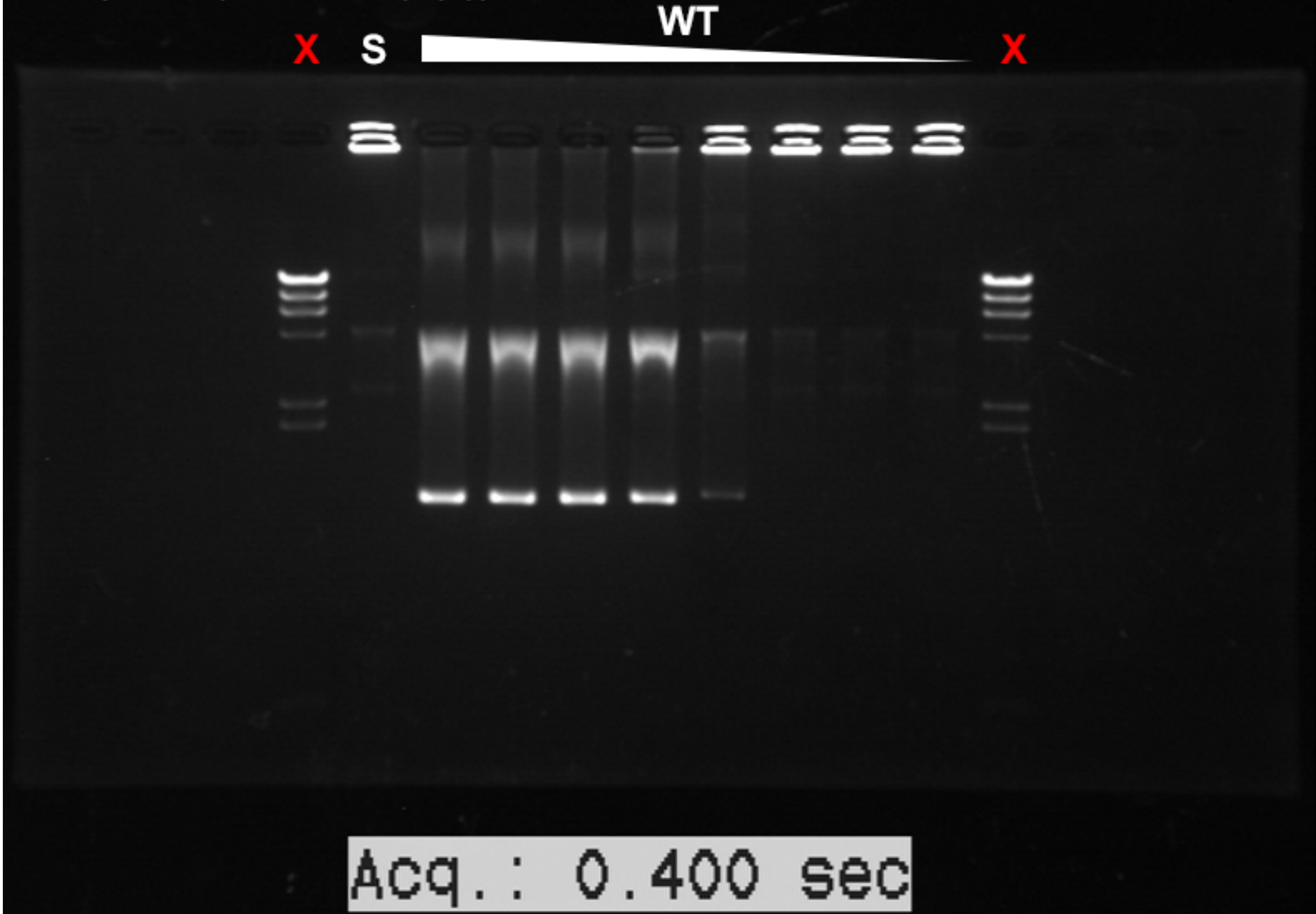

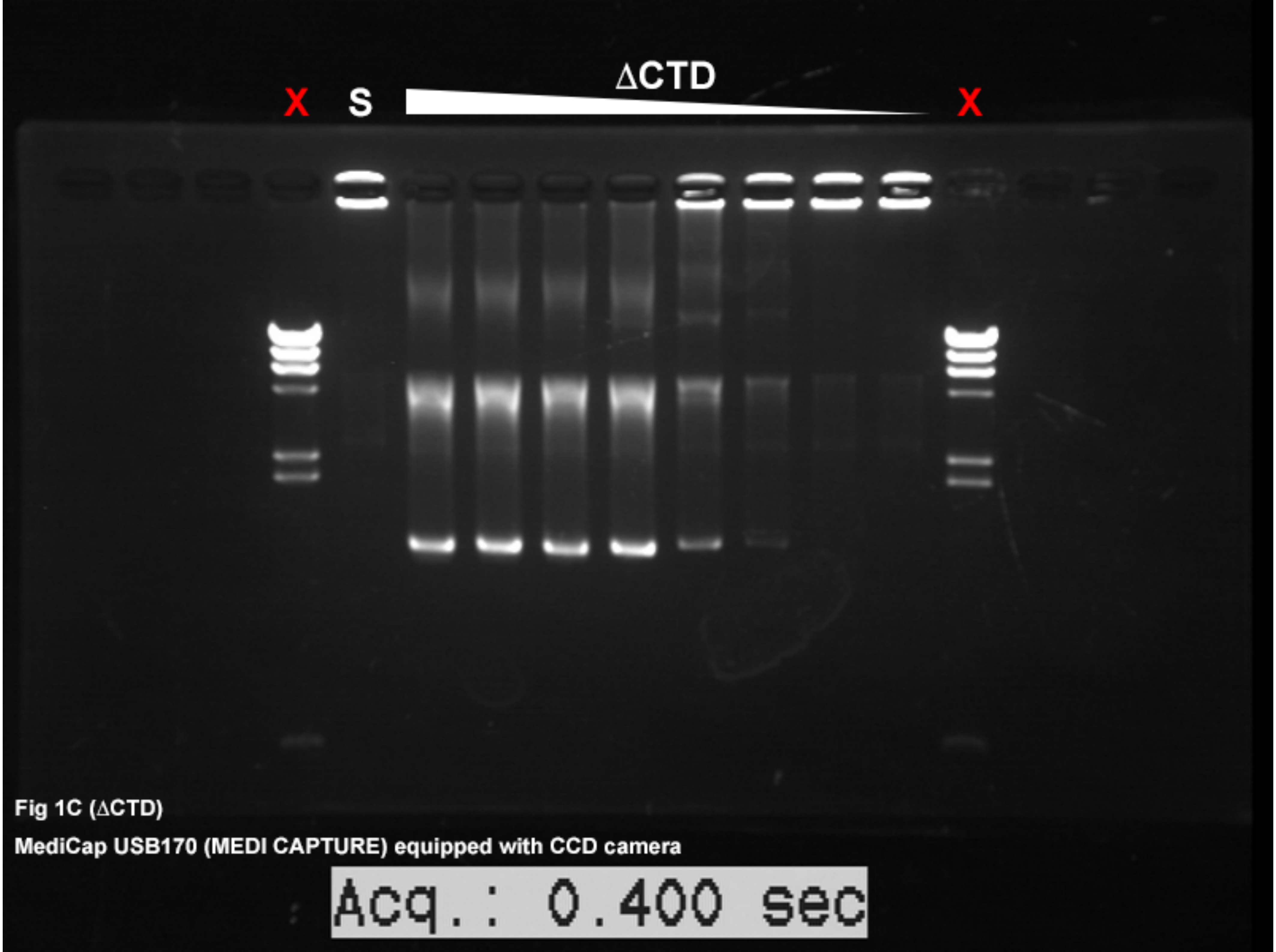

Fig 1C (ΔCTD)

MediCap USB170 (MEDI CAPTURE) equipped with CCD camera

Fig 1D (WT and ΔCTD)

MediCap USB170 (MEDI CAPTURE) equipped with CCD camera

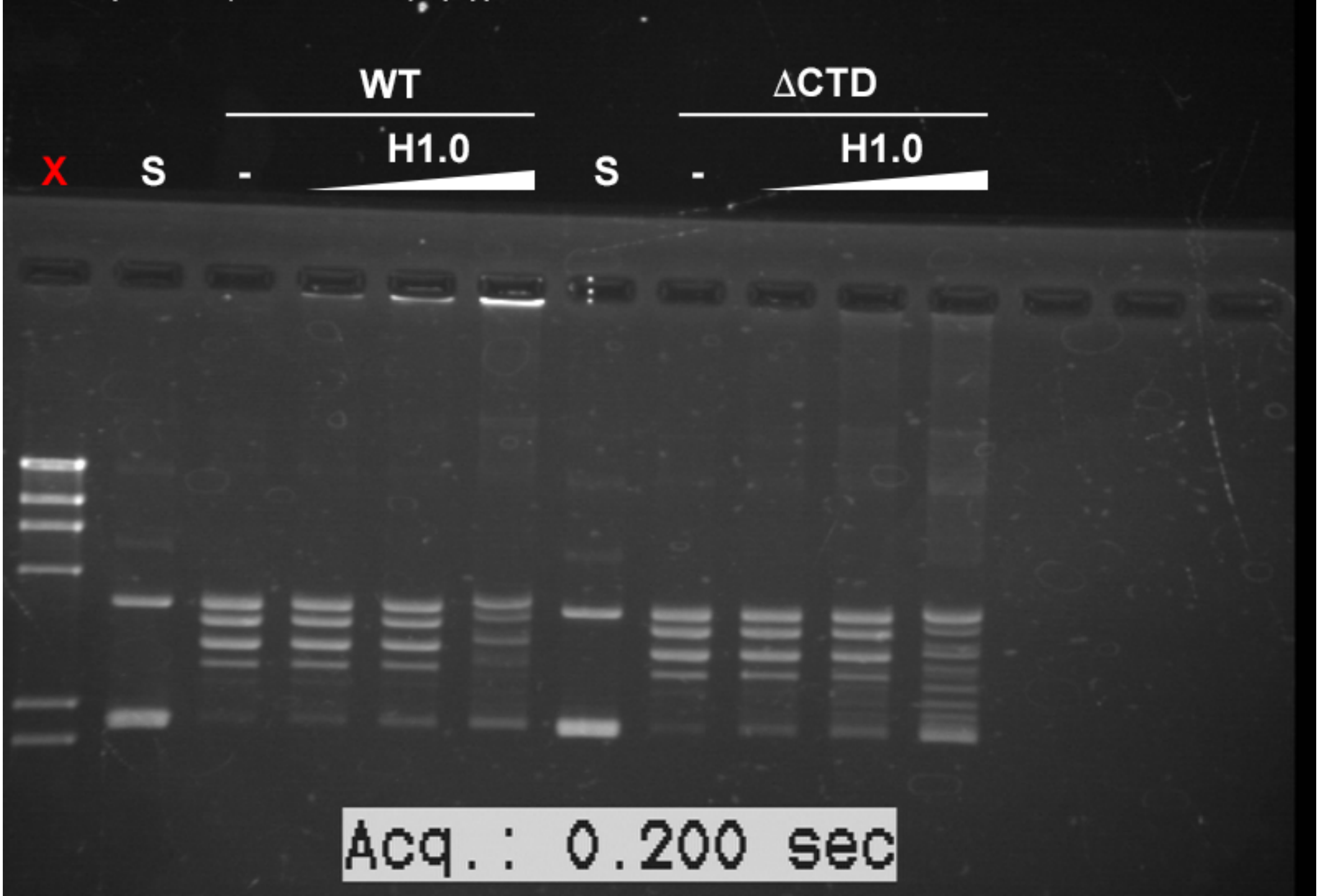

Fig 1E (WT, ΔCTD)

MediCap USB170 (MEDI CAPTURE) equipped with CCD camera

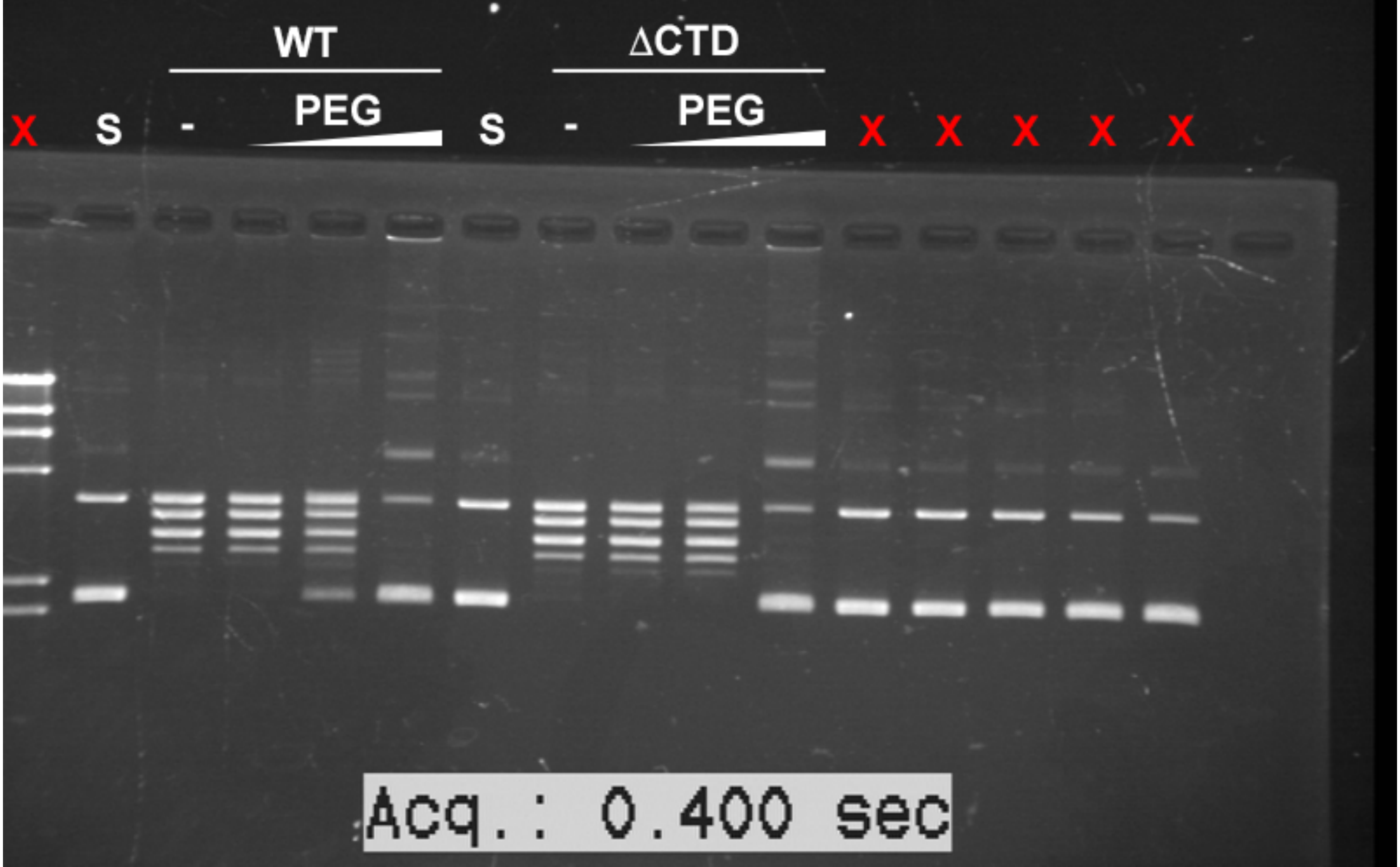

Fig 2B (1201-1614)

MediCap USB170 (MEDI CAPTURE) equipped with CCD camera

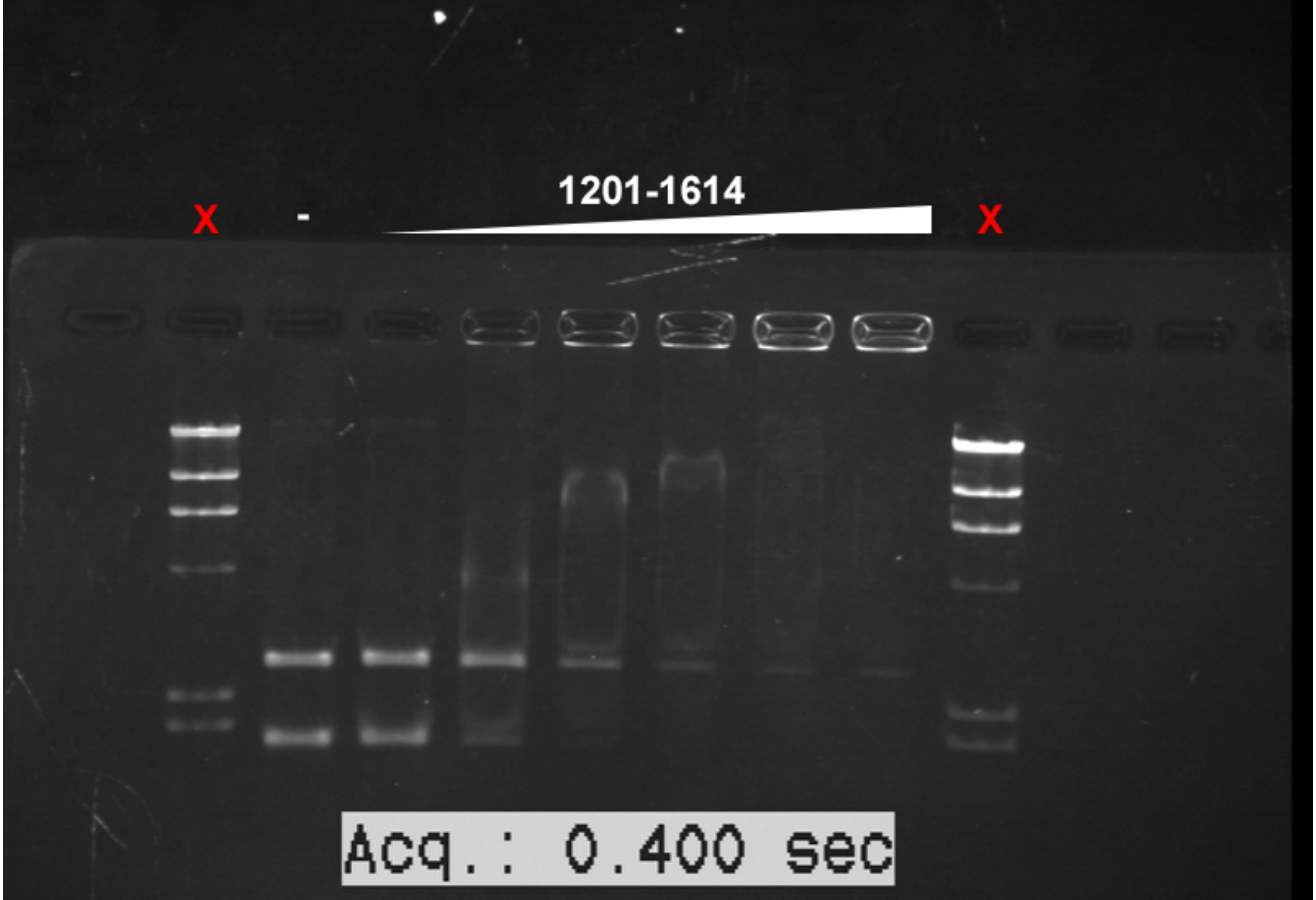

Fig 2B (1201-1320)

MediCap USB170 (MEDI CAPTURE) equipped with CCD camera

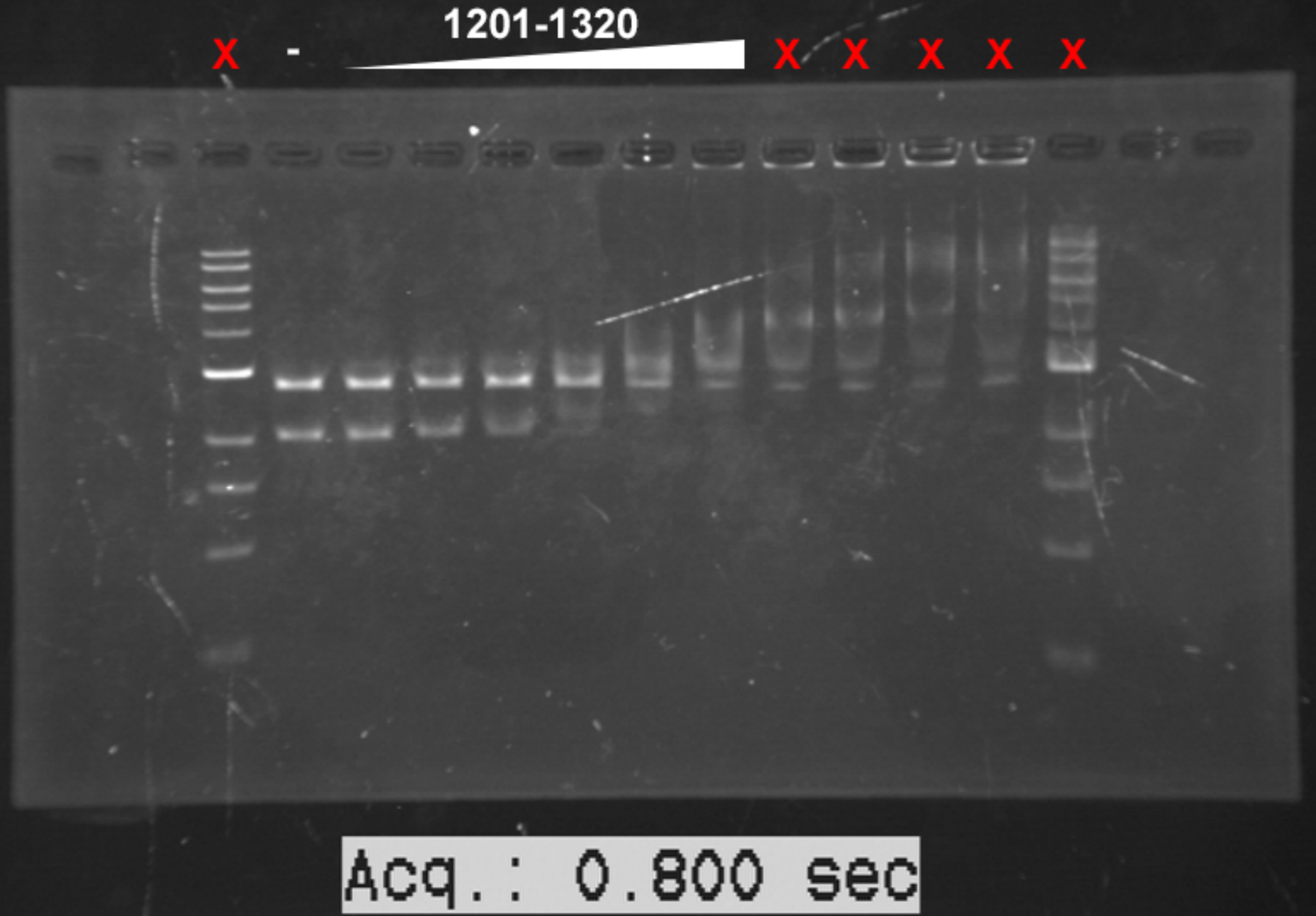

Fig 2B (1321-1614)

MediCap USB170 (MEDI CAPTURE) equipped with CCD camera

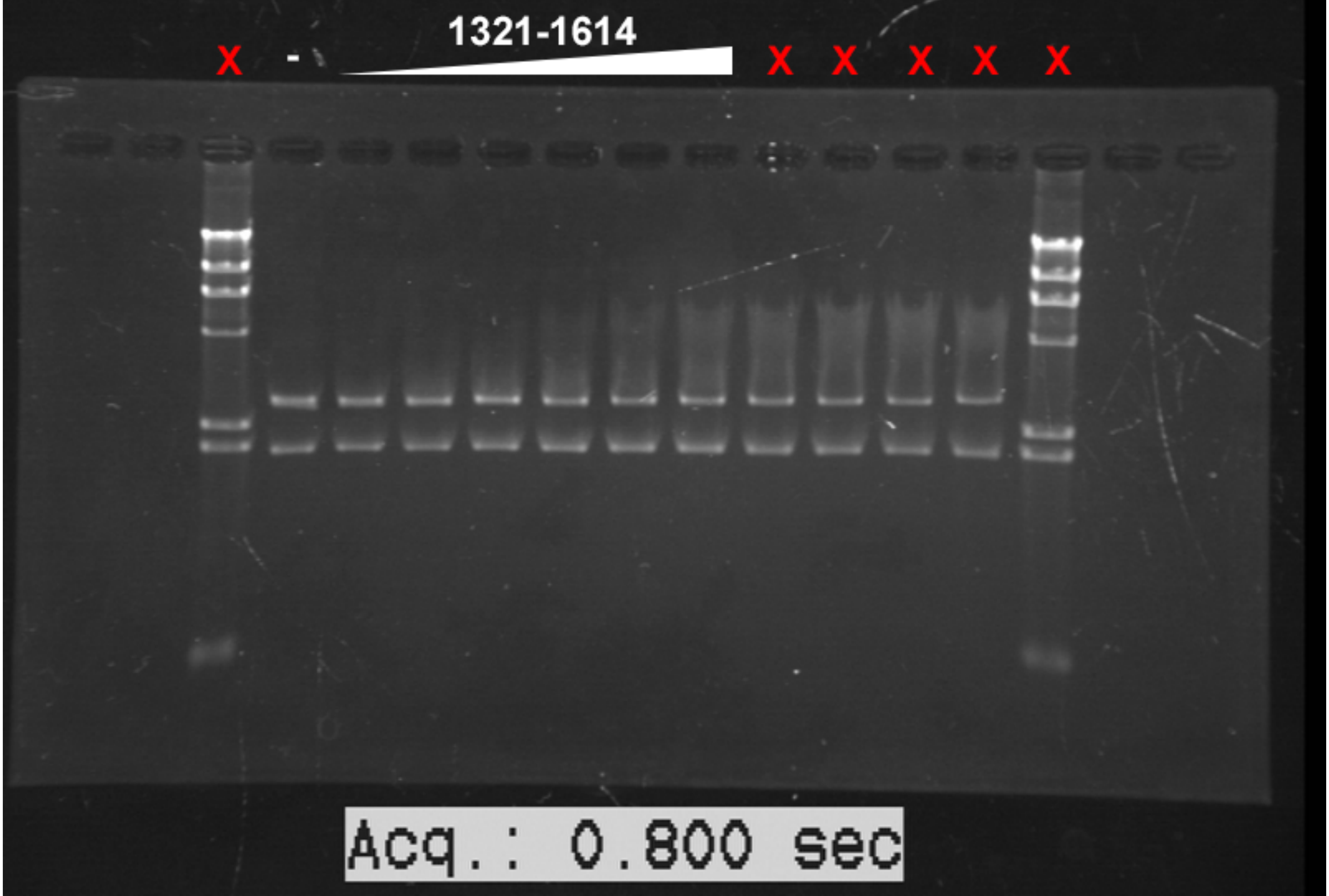

Fig 2C (1-1320)

MediCap (MEDI CAPTURE) equipped with CCD camera

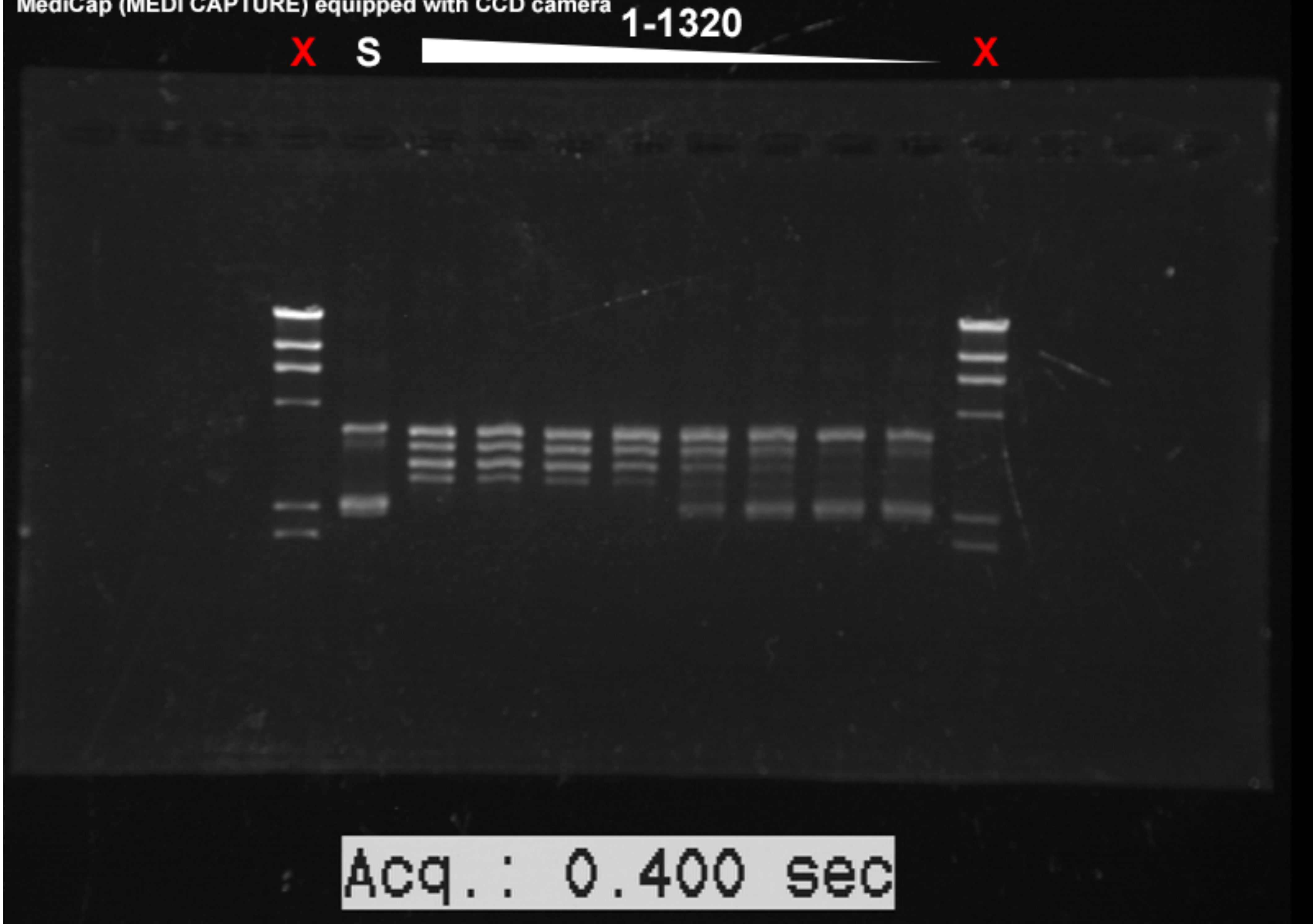

Fig 2C (Δ1201-1320)

MediCap (MEDI CAPTURE) equipped with CCD camera

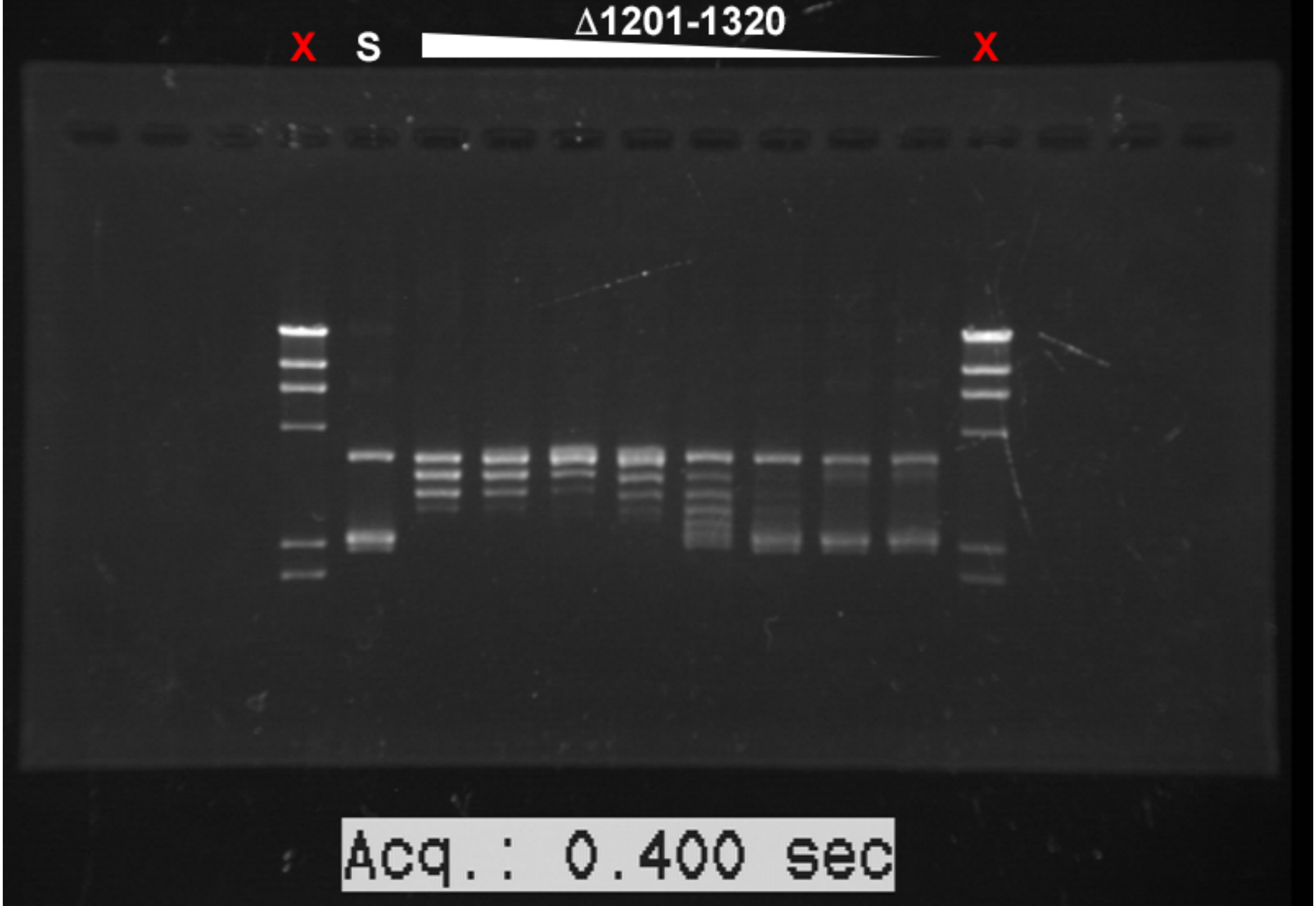

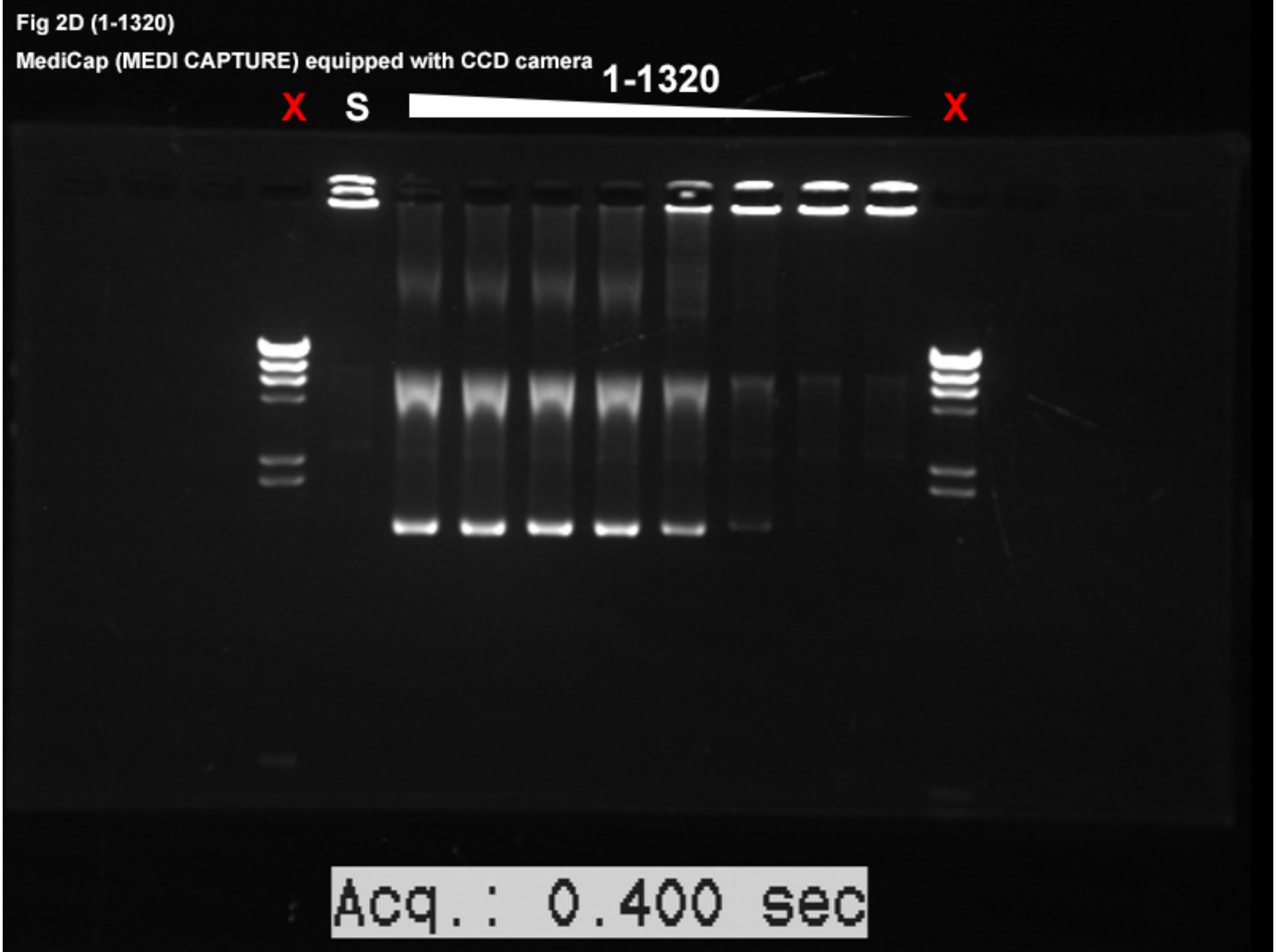
Fig 2D (1-1320)
MediCap (MEDI CAPTURE) equipped with CCD camera
1-1320
X
S
X
Acq.: 0.400 sec

Fig 2D (Δ1201-1320)

MediCap (MEDI CAPTURE) equipped with CCD camera

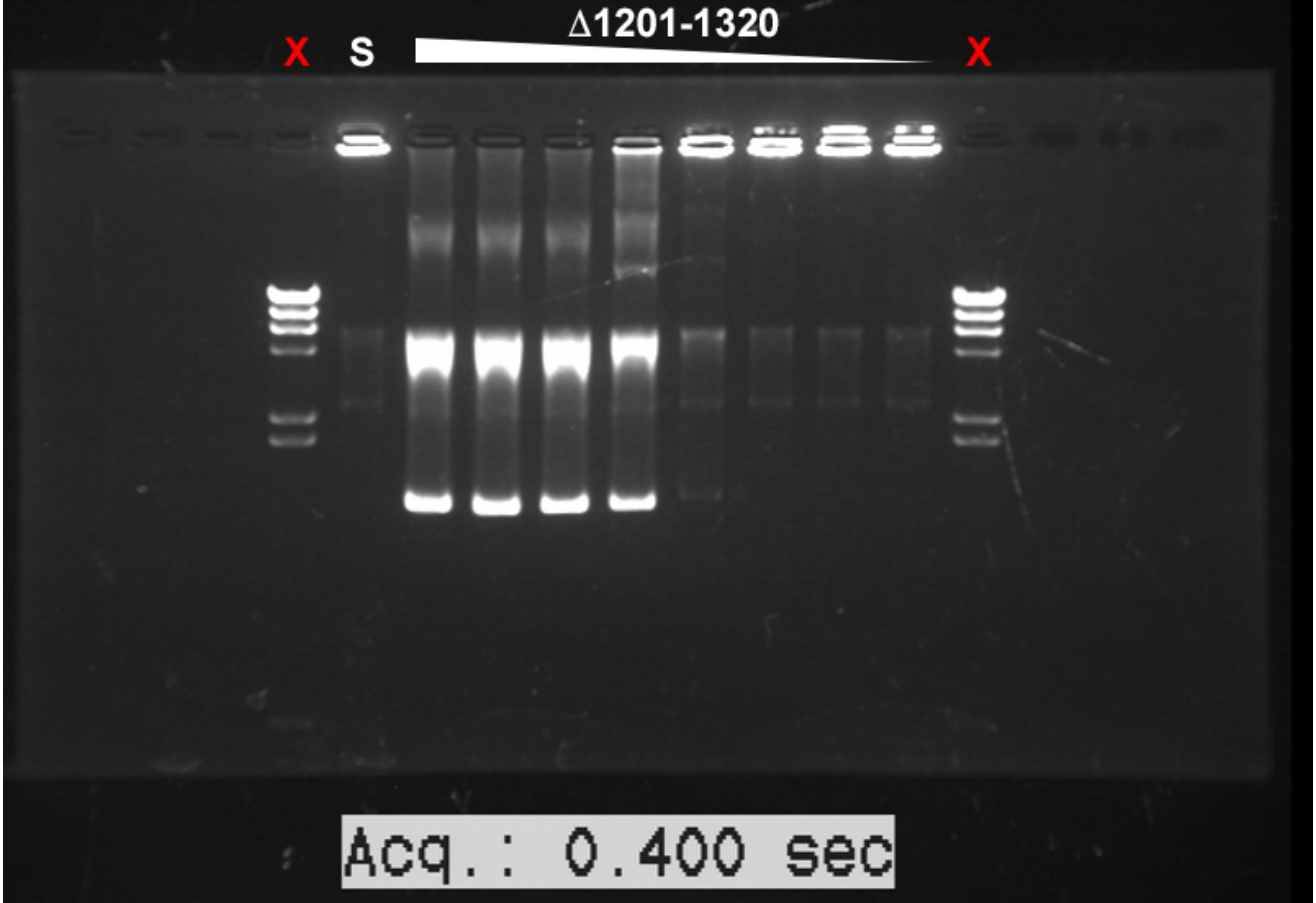

Fig 2E (1-1320 and Δ1201-1320)

MediCap USB170 (MEDI CAPTURE) equipped with CCD camera

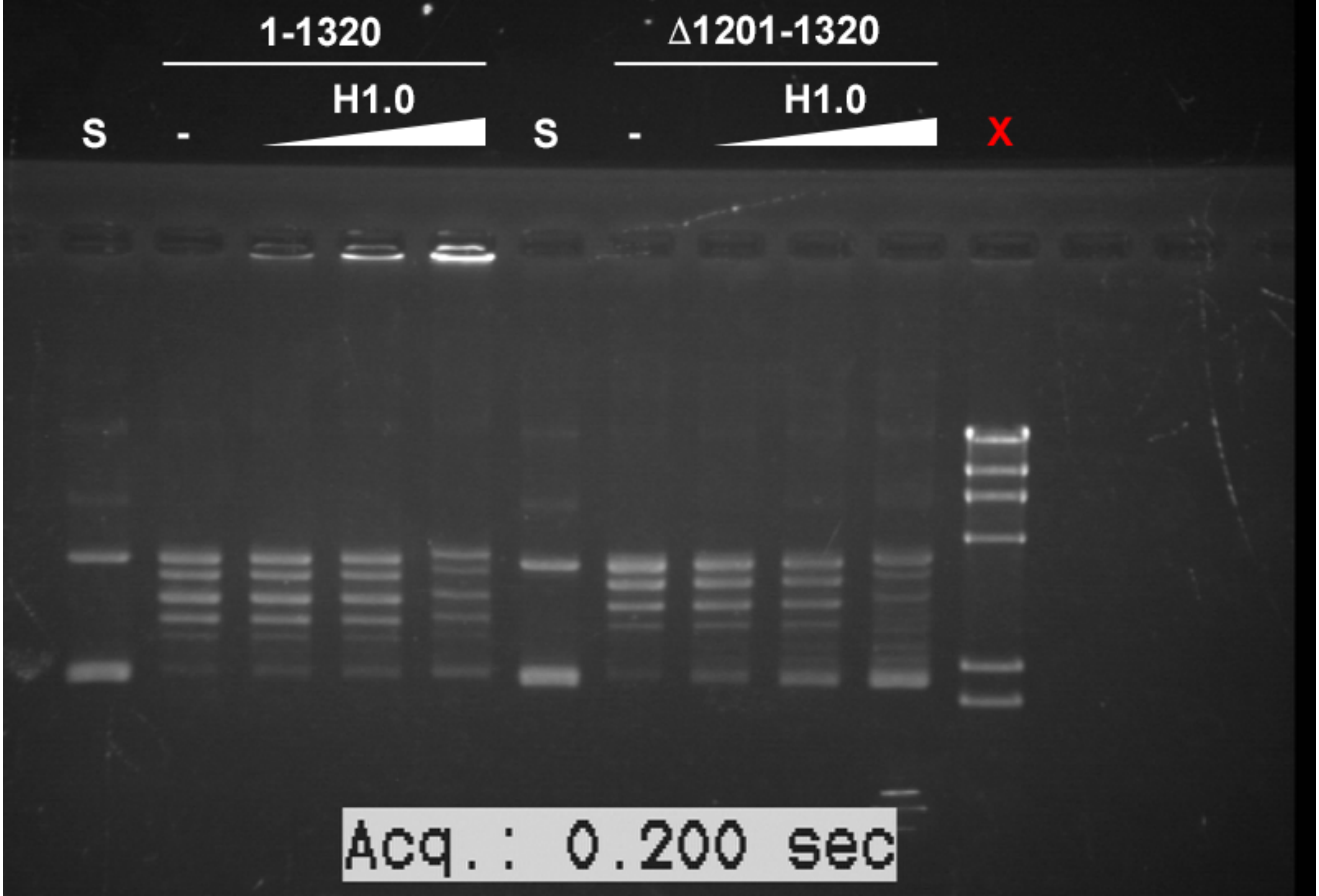

Fig 2F (1-1320 and Δ1201-1320)

MediCap USB170 (MEDI CAPTURE) equipped with CCD camera

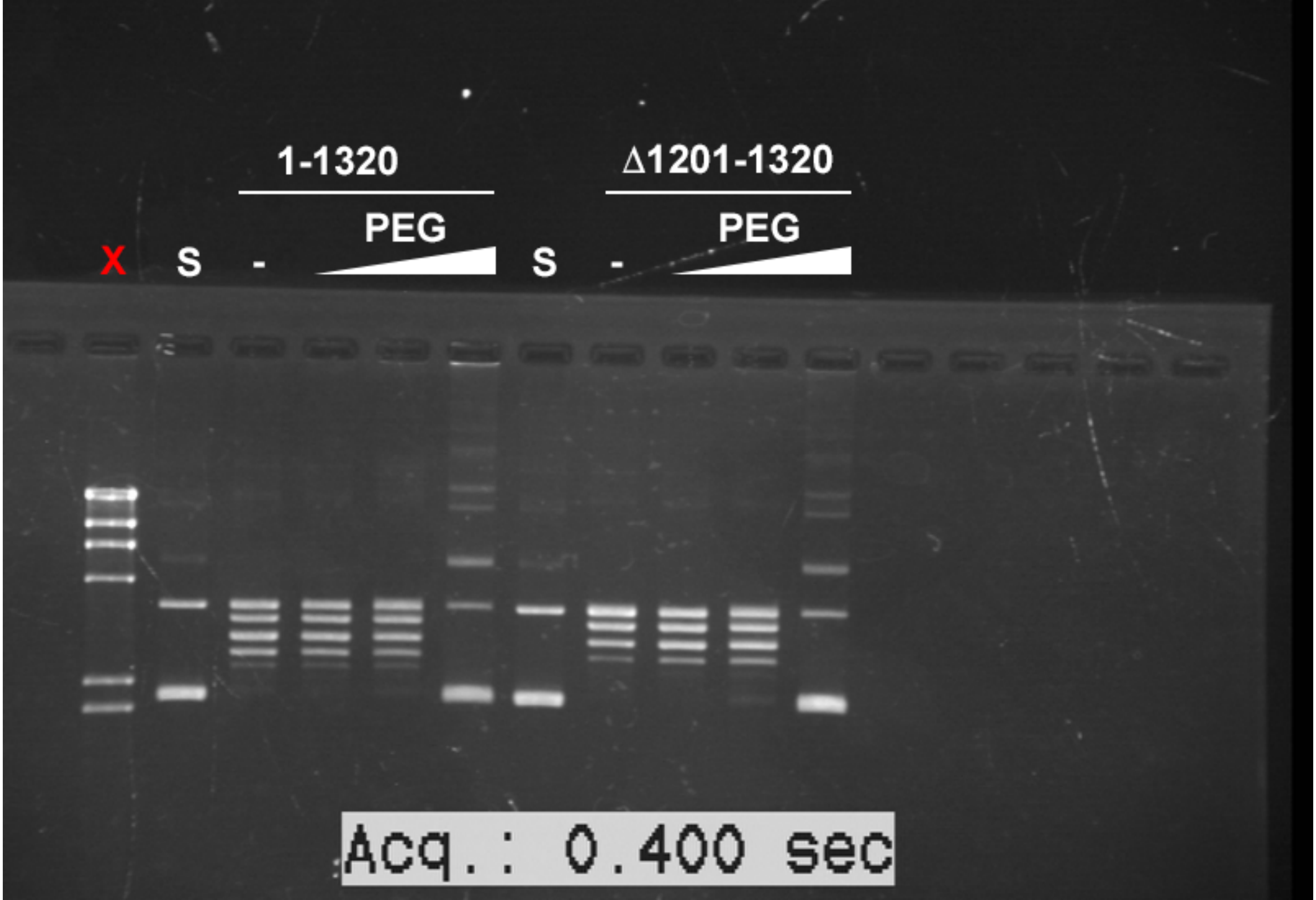

Fig 3A

MediCap USB170 (MEDI CAPTURE) equipped with CCD camera

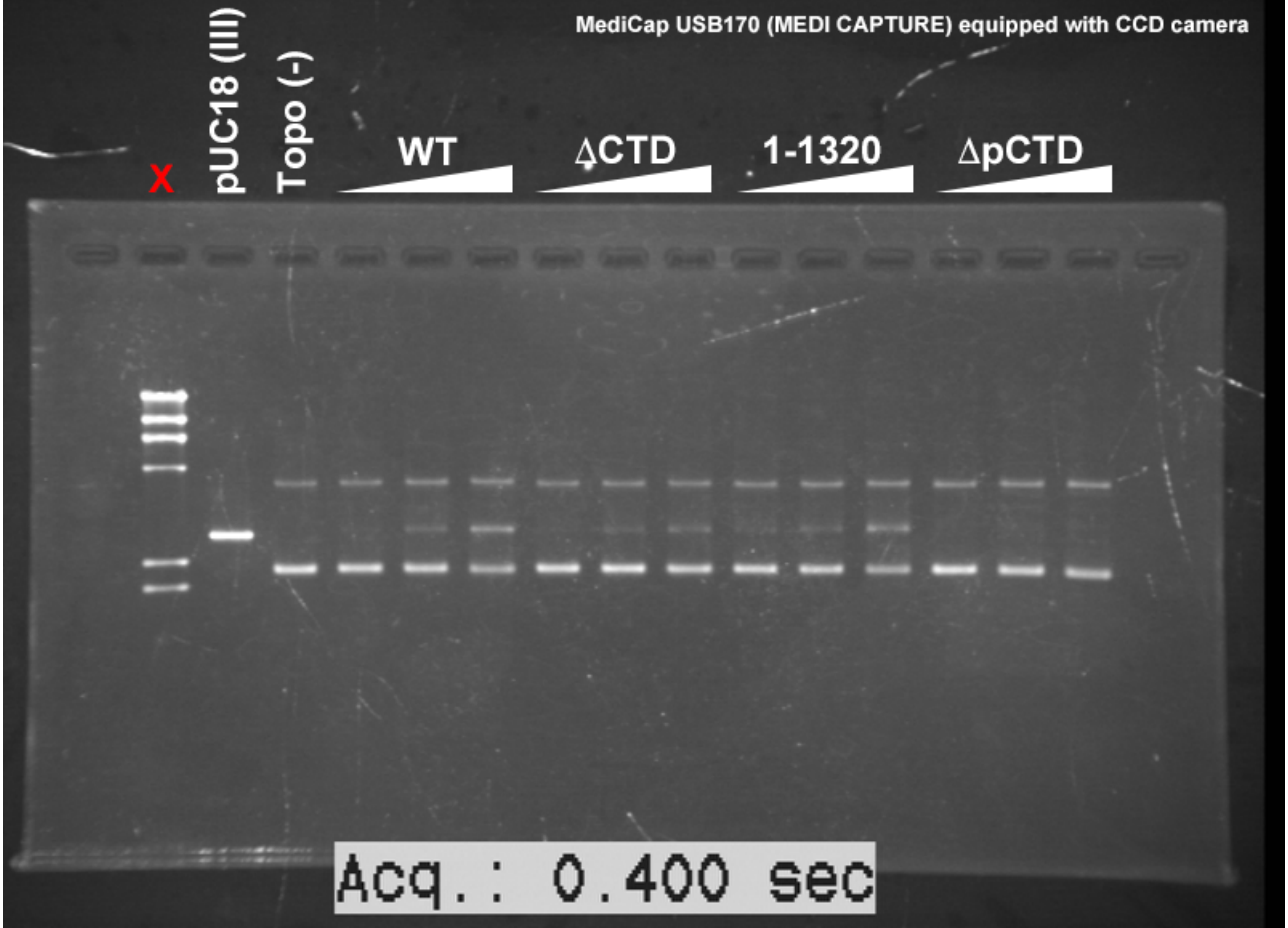

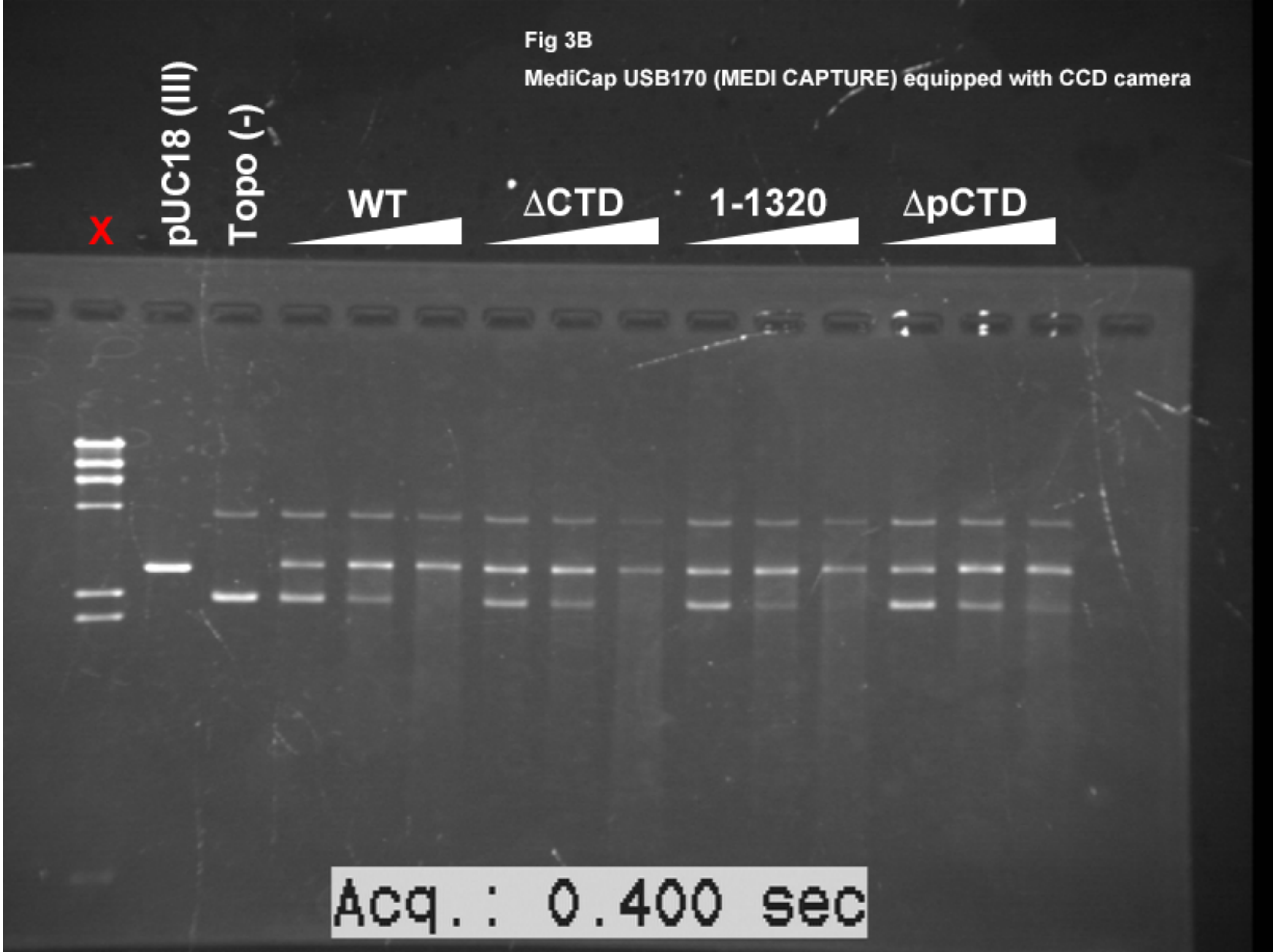

Fig 3B
MediCap USB170 (MEDI CAPTURE) equipped with CCD camera
X
pUC18 (III)
Topo (-)
WT
ΔCTD
1-1320
ΔpCTD
Acq. : 0.400 sec

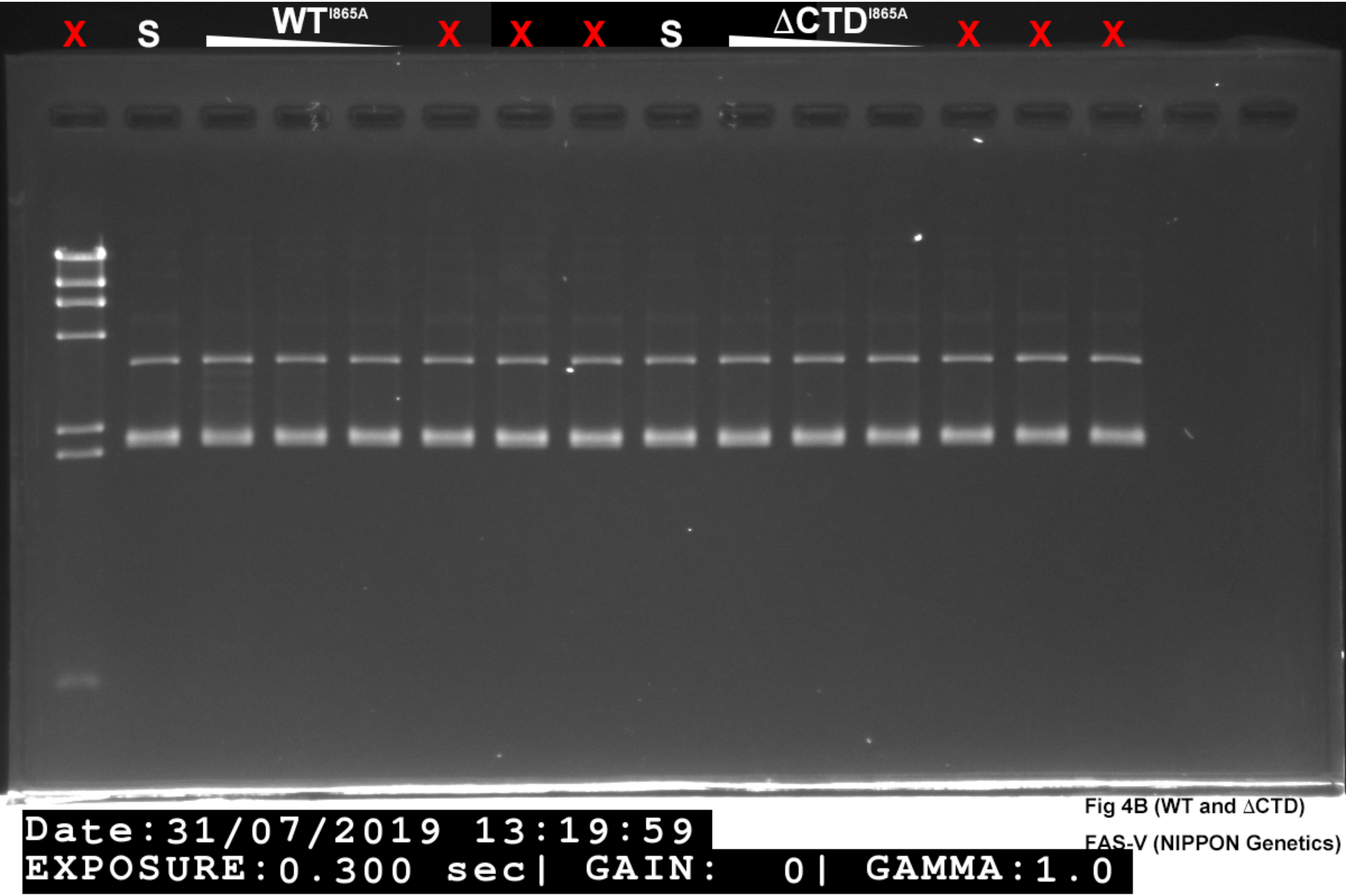

Fig 4B (WT and ΔCTD)

FAS-V (NIPPON Genetics)

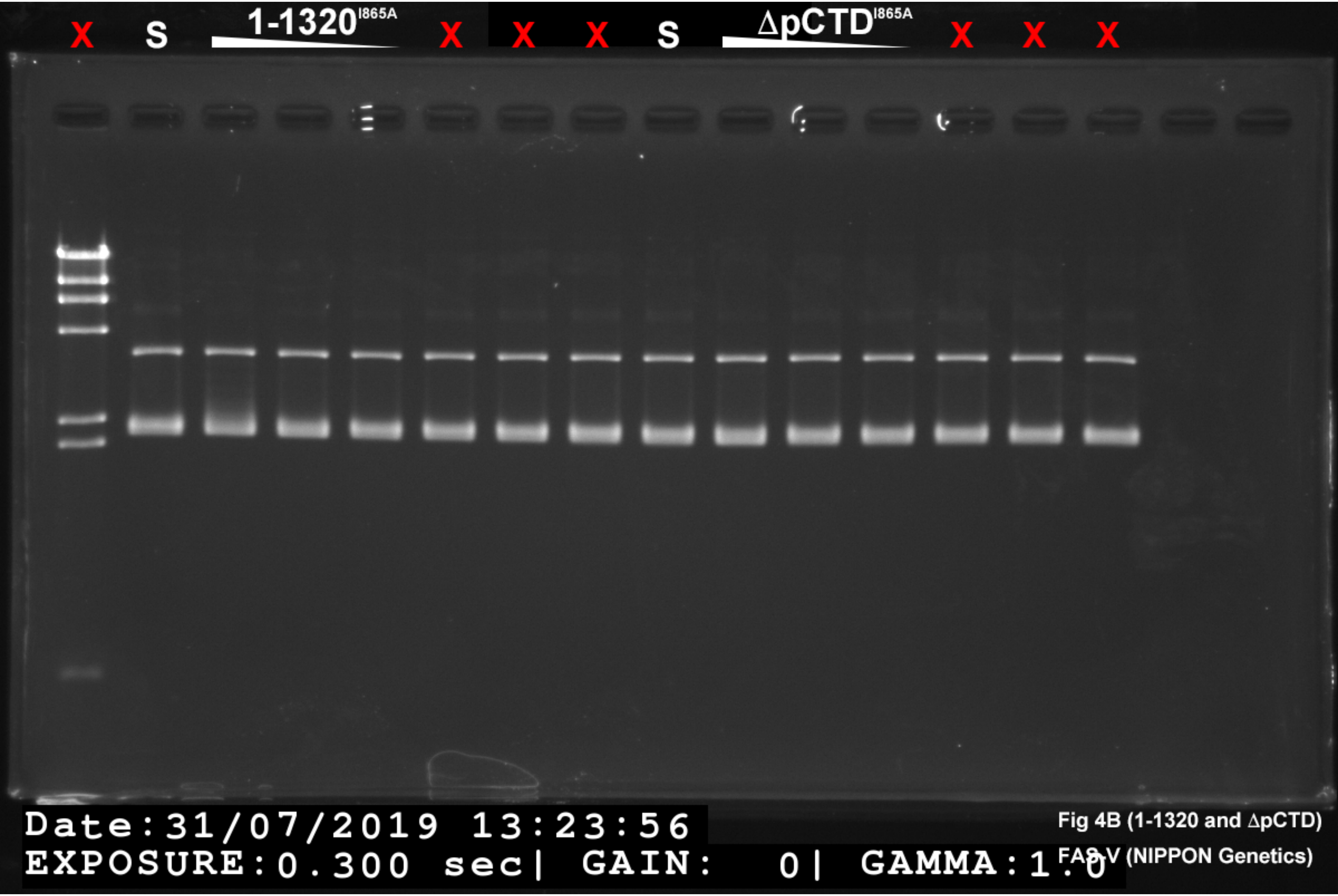
X
S
1-1320 I865A
X
X
X
S
ΔpCTD I865A
X
X
X
Date:31/07/2019 13:23:56
EXPOSURE:0.300 sec| GAIN: 0| GAMMA:1.0
Fig 4B (1-1320 and ΔpCTD)
FAS-V (NIPPON Genetics)

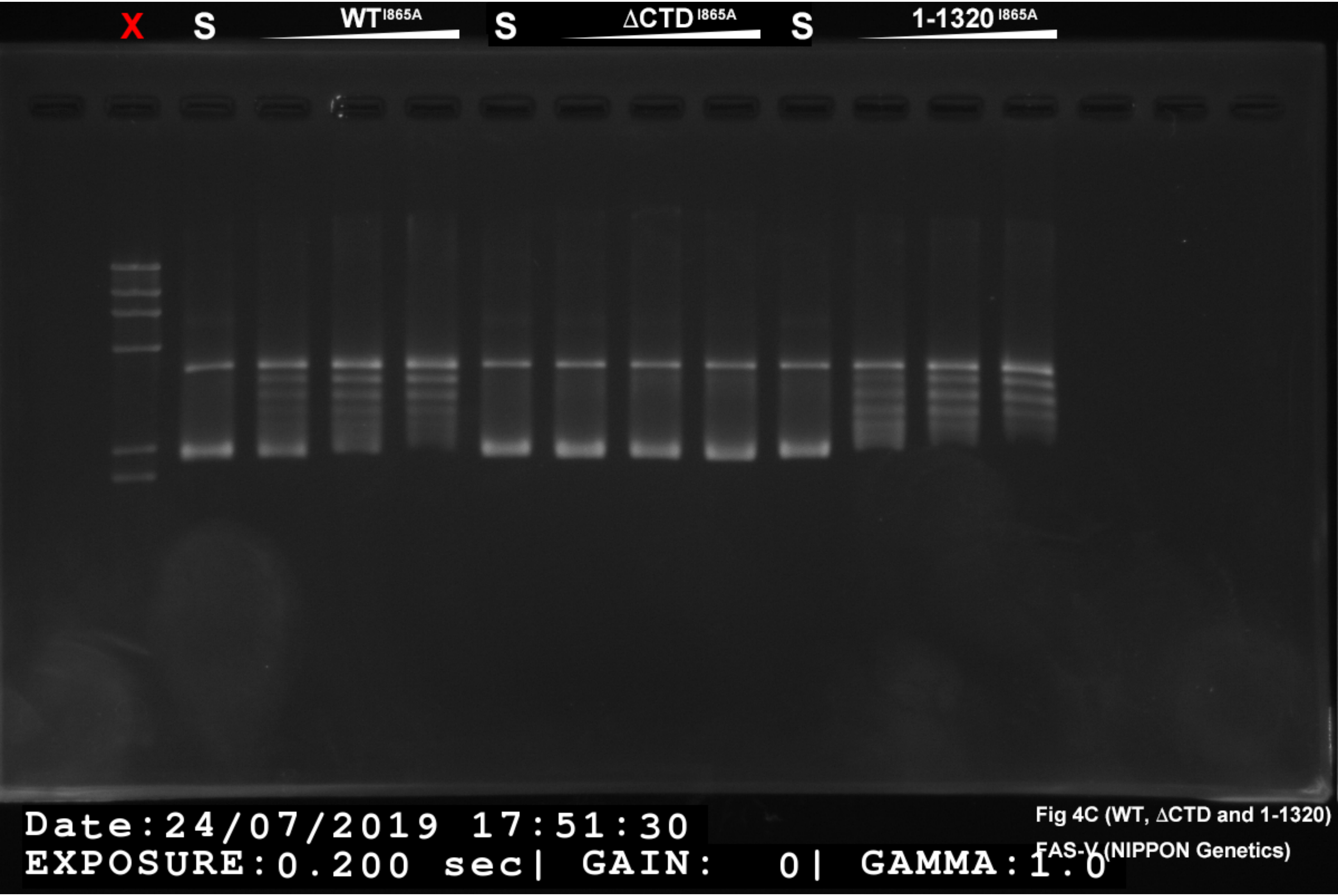
X
S
WT I865A
S
ΔCTD I865A
S
1-1320 I865A
Date:24/07/2019 17:51:30
EXPOSURE:0.200 sec| GAIN: 0| GAMMA:1.0
Fig 4C (WT, ΔCTD and 1-1320)
FAS-V (NIPPON Genetics)

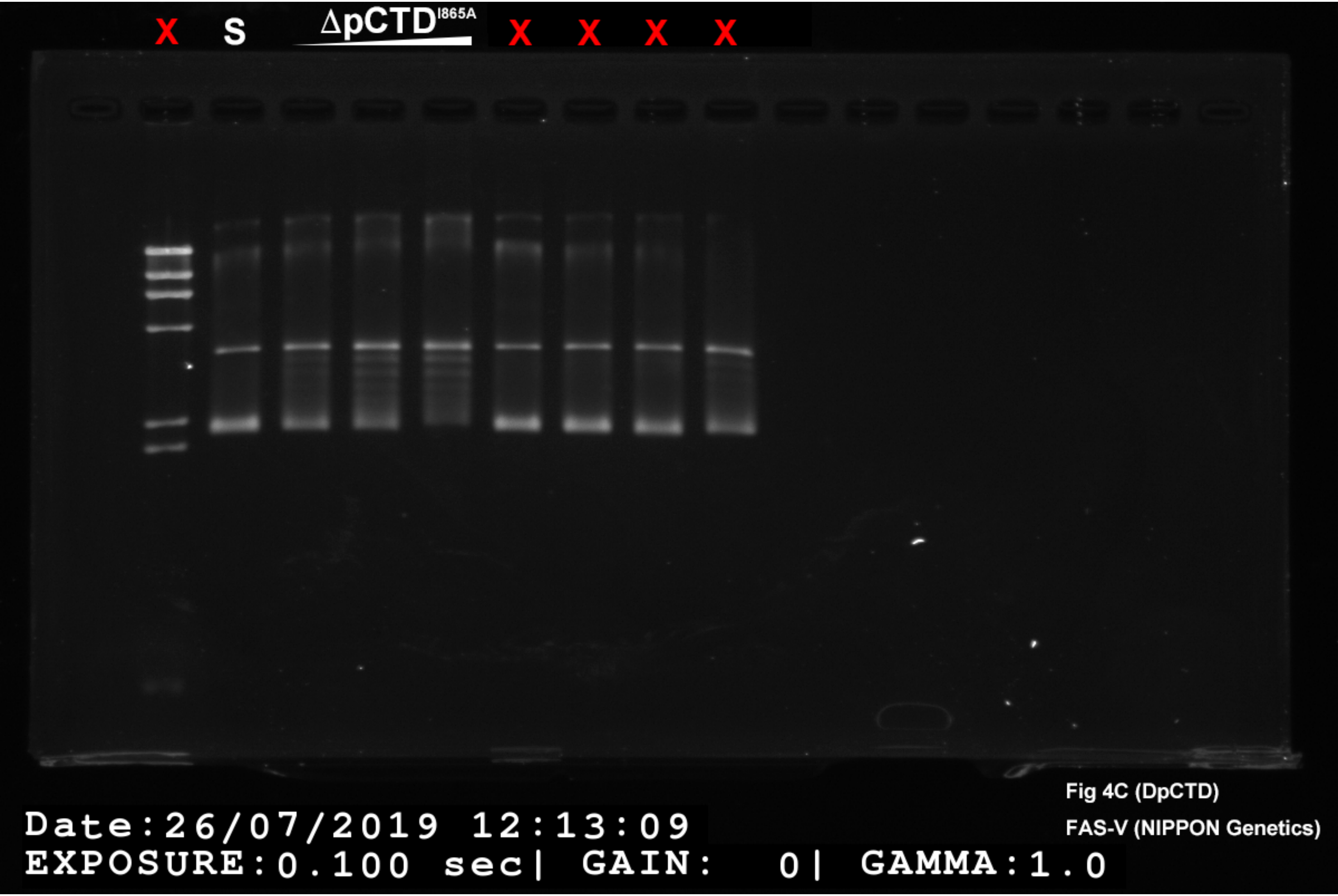
X
S
ΔpCTD I865A
X
X
X
X
Fig 4C (DpCTD)
FAS-V (NIPPON Genetics)
Date:26/07/2019 12:13:09
EXPOSURE:0.100 sec| GAIN: 0| GAMMA:1.0

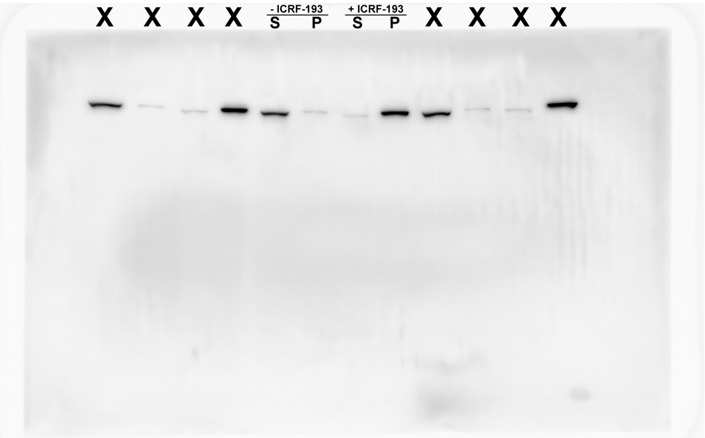

Fig 6B (WT), LAS4000 mini (GE Healthcare)

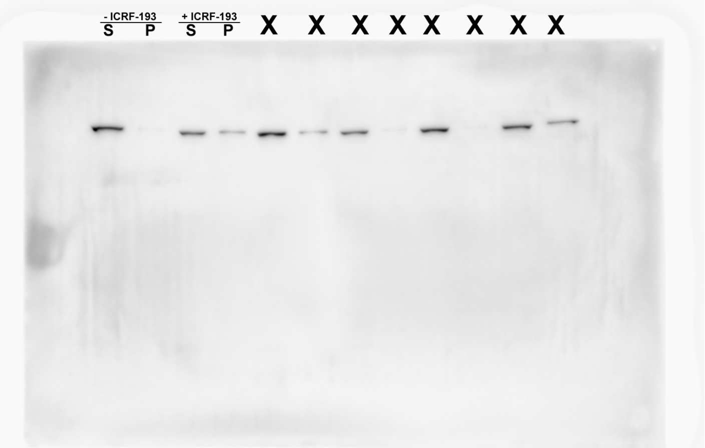

Fig 6B (∆pCTD), LAS 4000mini (GE Healthcare)

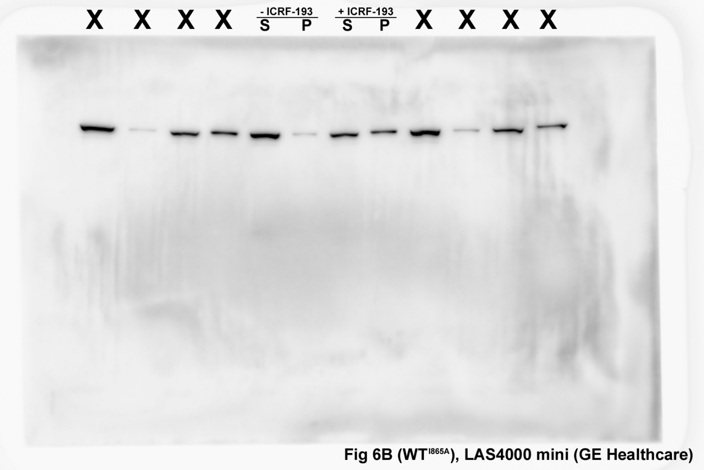

Fig 6B (WT^{I865A}), LAS4000 mini (GE Healthcare)

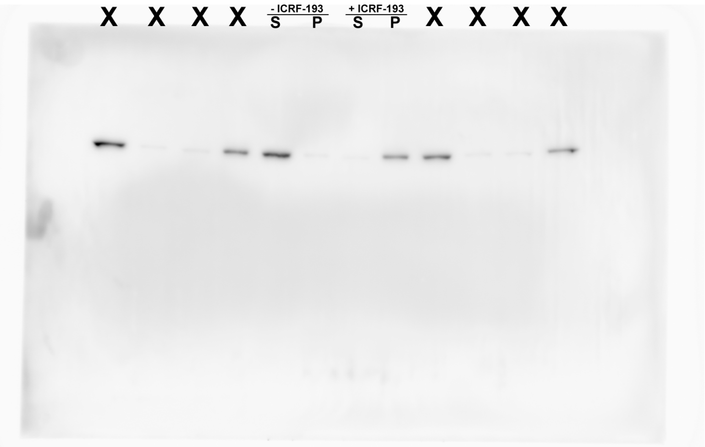

Fig 6B (ΔpCTD^{I865A}), LAS 4000mini (GE Healthcare)

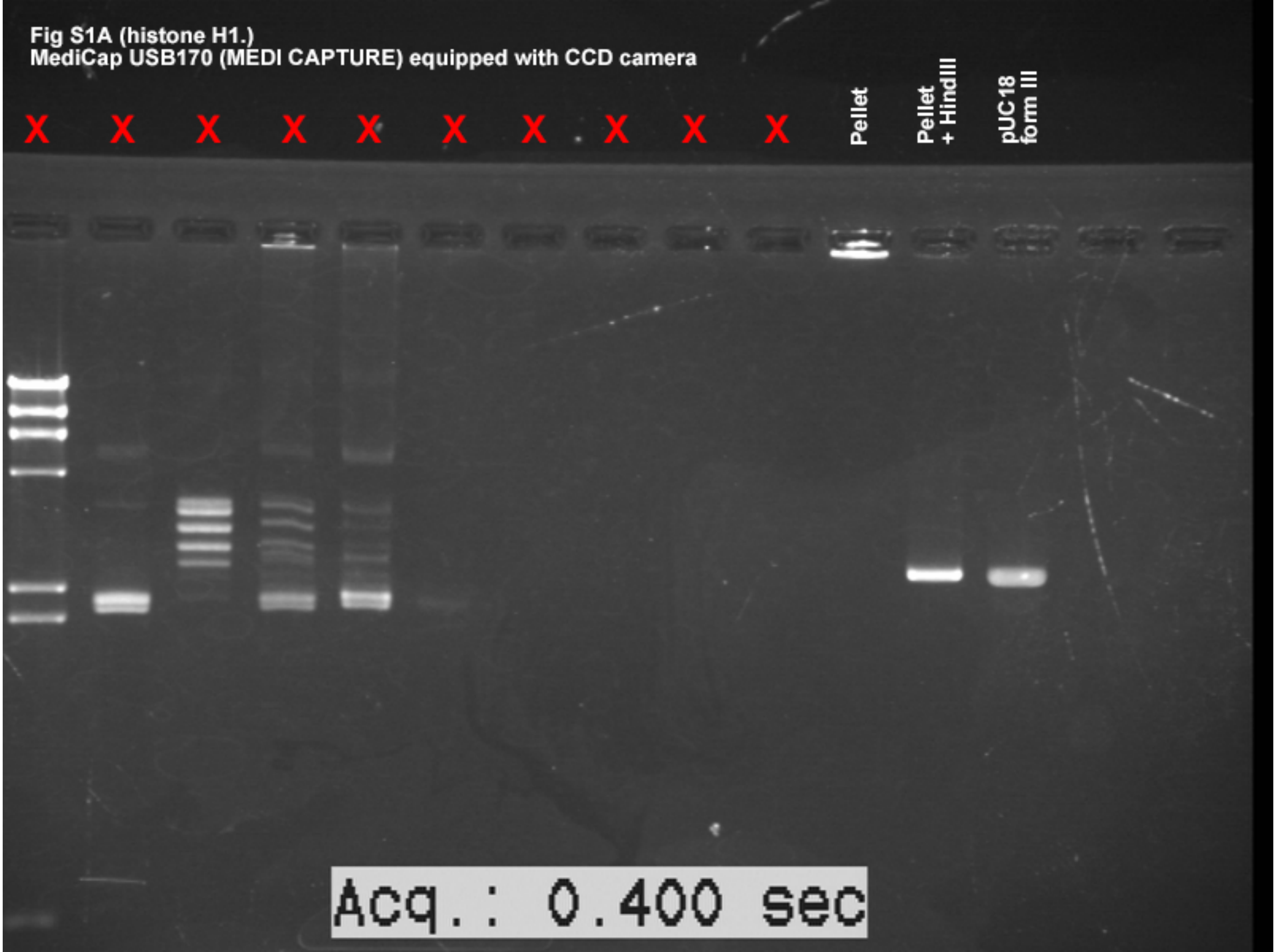
Fig S1A (histone H1.)
MediCap USB170 (MEDI CAPTURE) equipped with CCD camera
X X X X X X X X X X
Pellet
Pellet + HindIII
pUC18 form III
Acq.: 0.400 sec

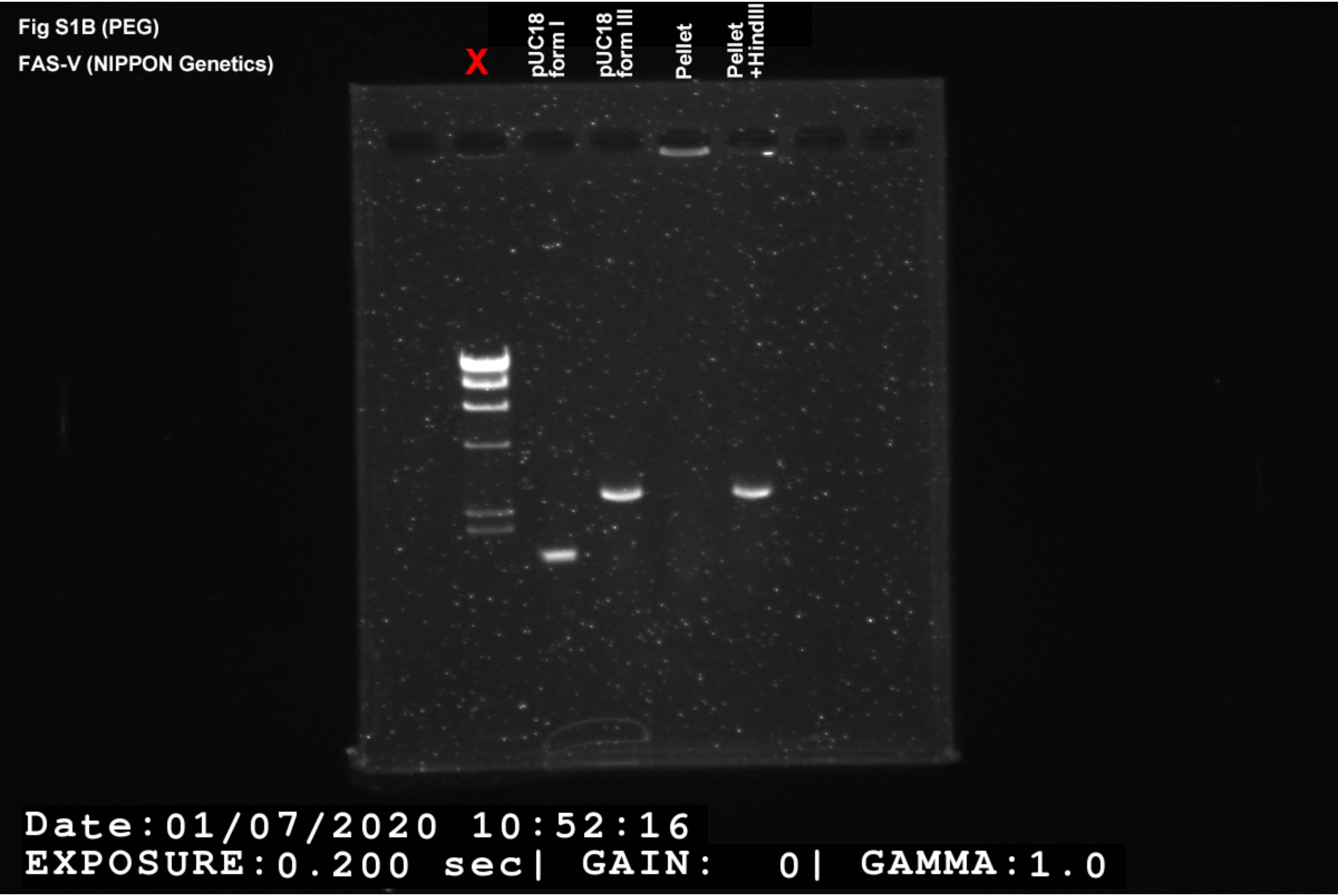
Fig S1B (PEG)
FAS-V (NIPPON Genetics)
X
pUC18 form I
pUC18 form III
Pellet
Pellet +HindIII
Date:01/07/2020 10:52:16
EXPOSURE:0.200 sec| GAIN: 0| GAMMA:1.0

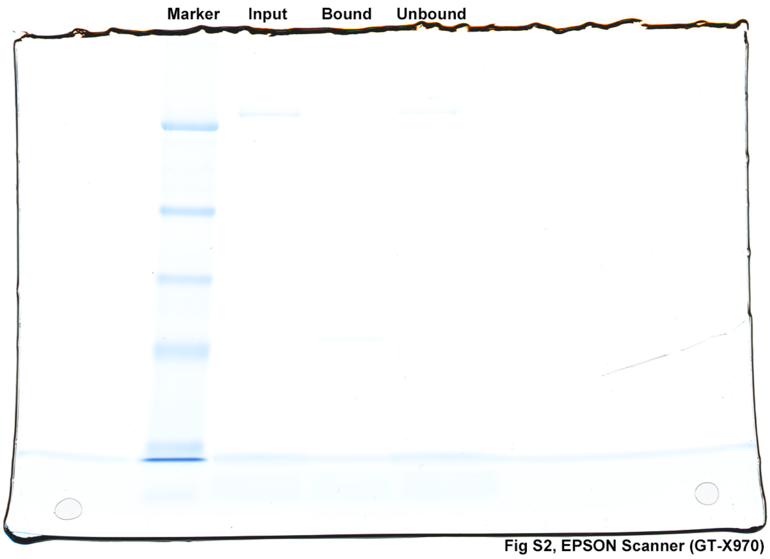

Fig S2, EPSON Scanner (GT-X970)

Fig S3B (ΔCTD' and ΔCRD)

MediCap USB170 (MEDI CAPTURE) equipped with CCD camera

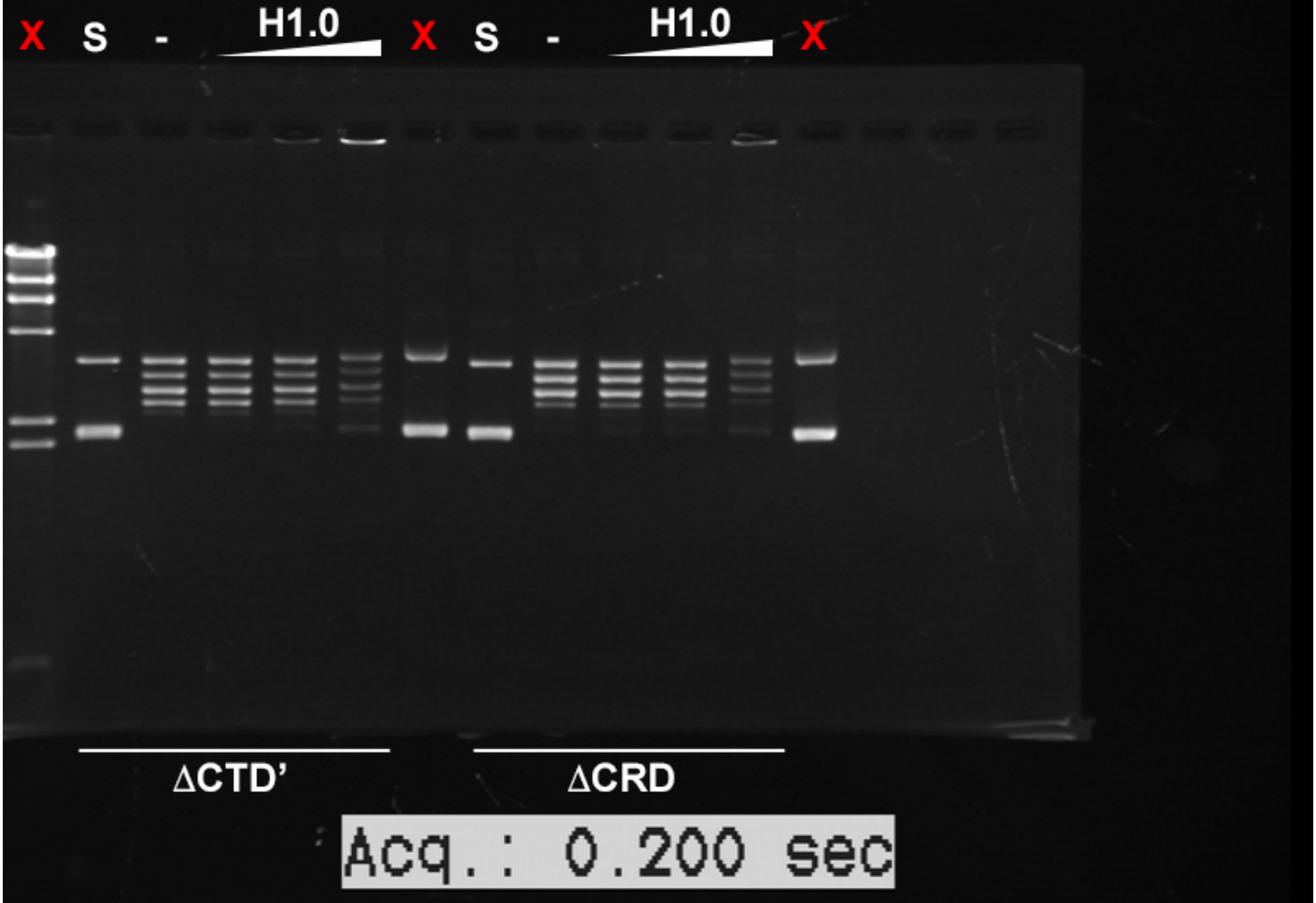

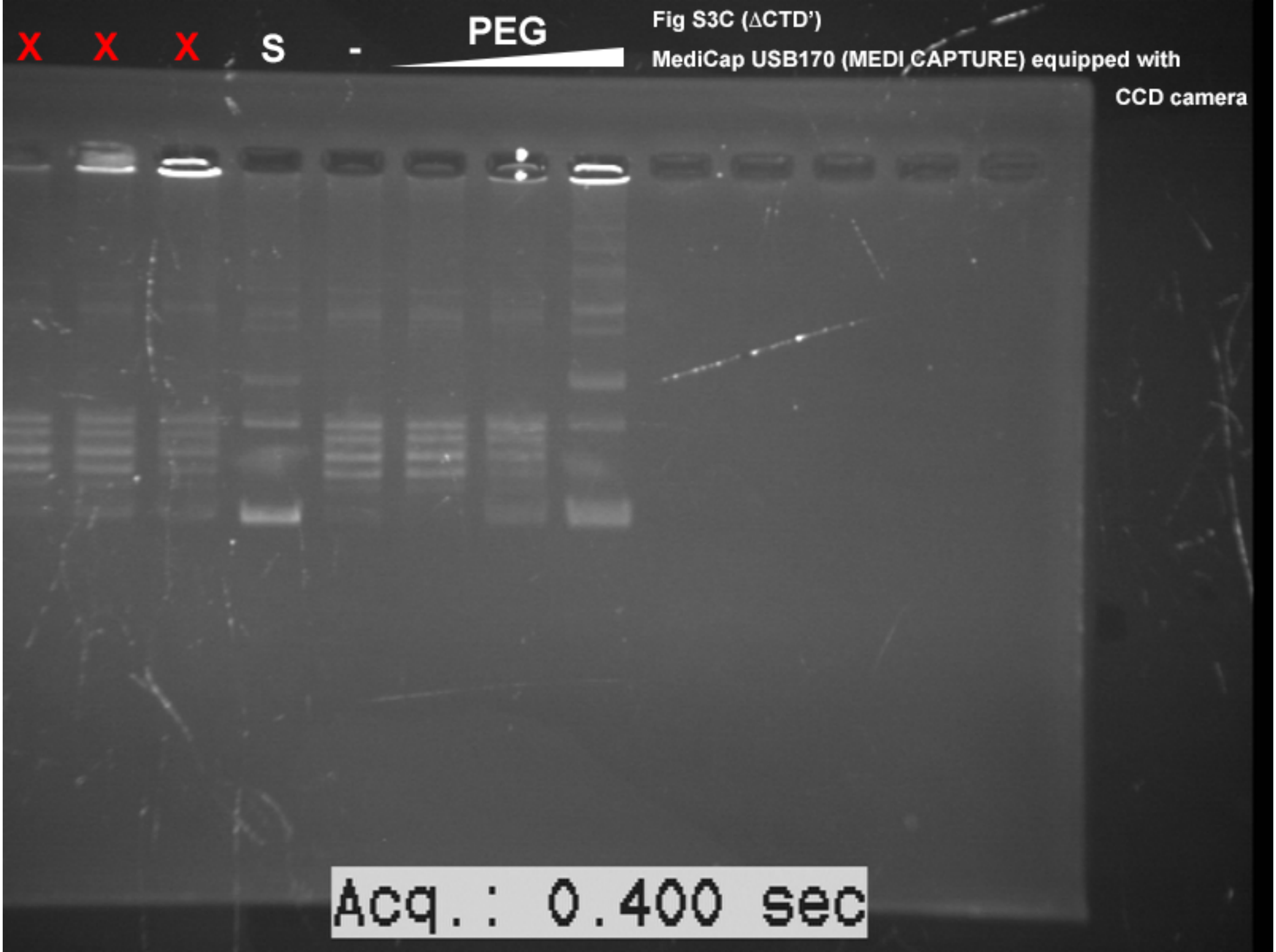
X
X
X
S
-
PEG
Fig S3C (ΔCTD')
MediCap USB170 (MEDI CAPTURE) equipped with
CCD camera
Acq.: 0.400 sec

Fig S3C (ΔCRD)

MediCap USB170 (MEDI CAPTURE) equipped with CCD camera

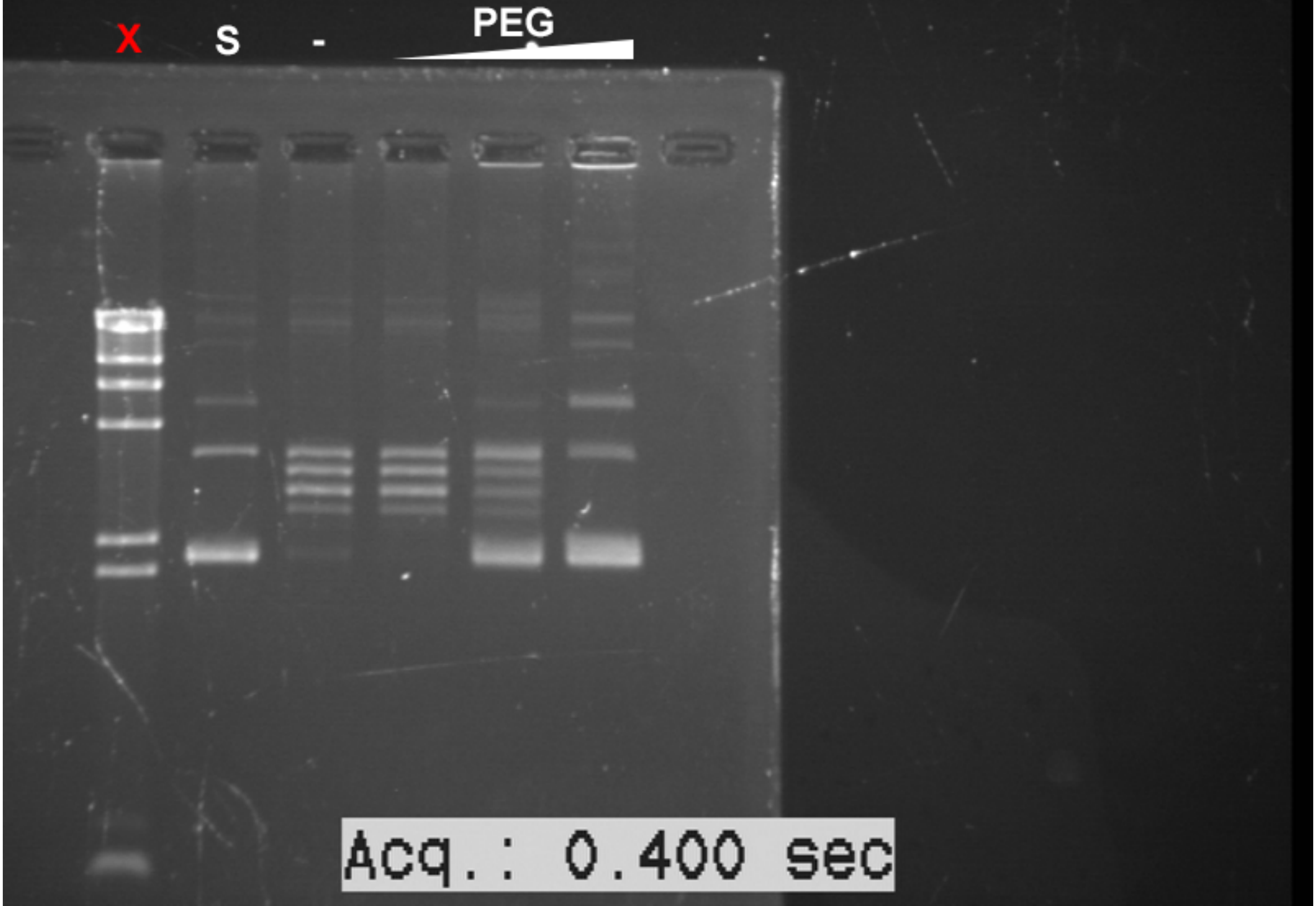

Fig S5B (WT)

MediCap USB170 (MEDI CAPTURE) equipped with CCD camera

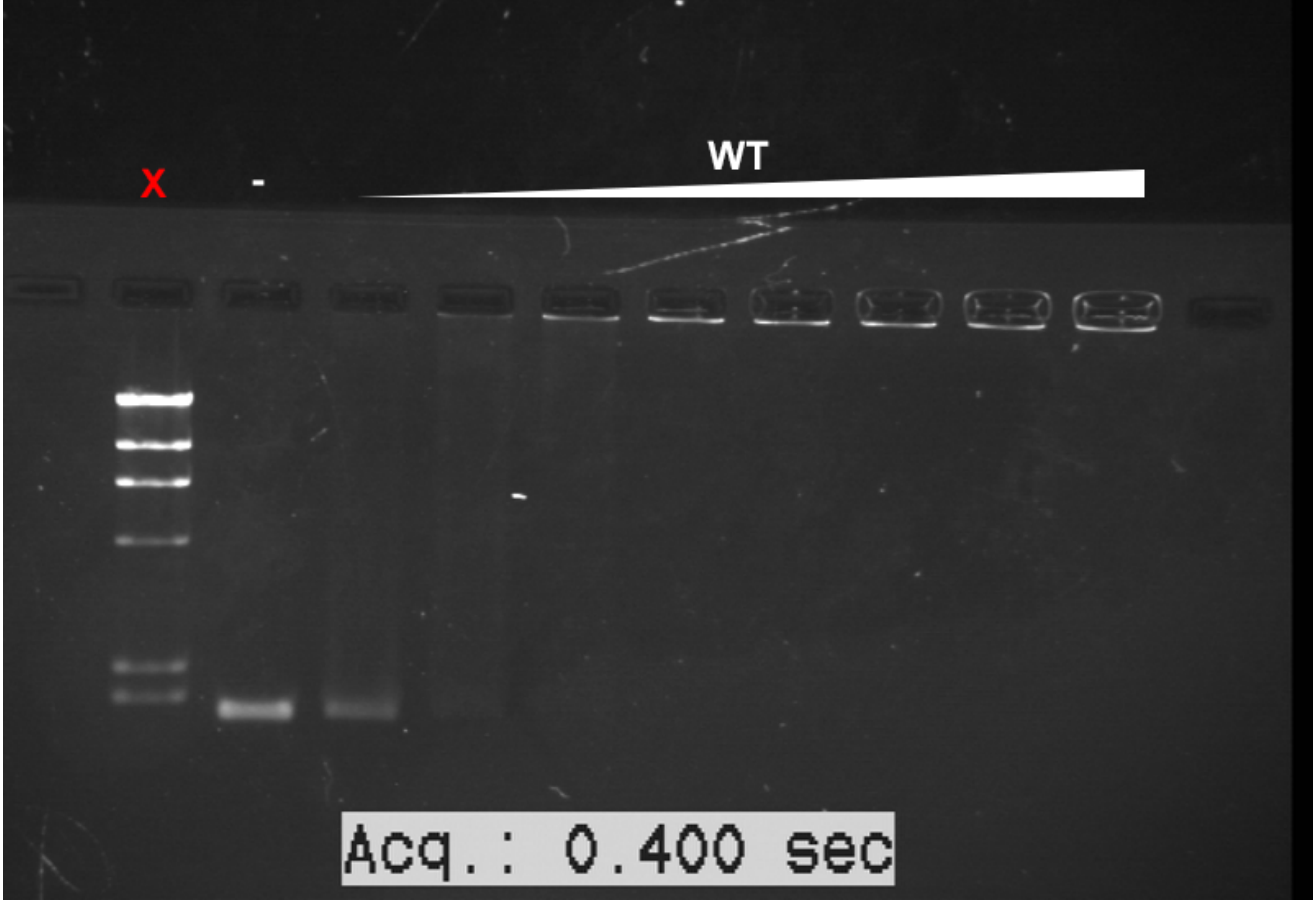

Fig S5B (ΔCTD)

MediCap USB170 (MEDI CAPTURE) equipped with CCD camera

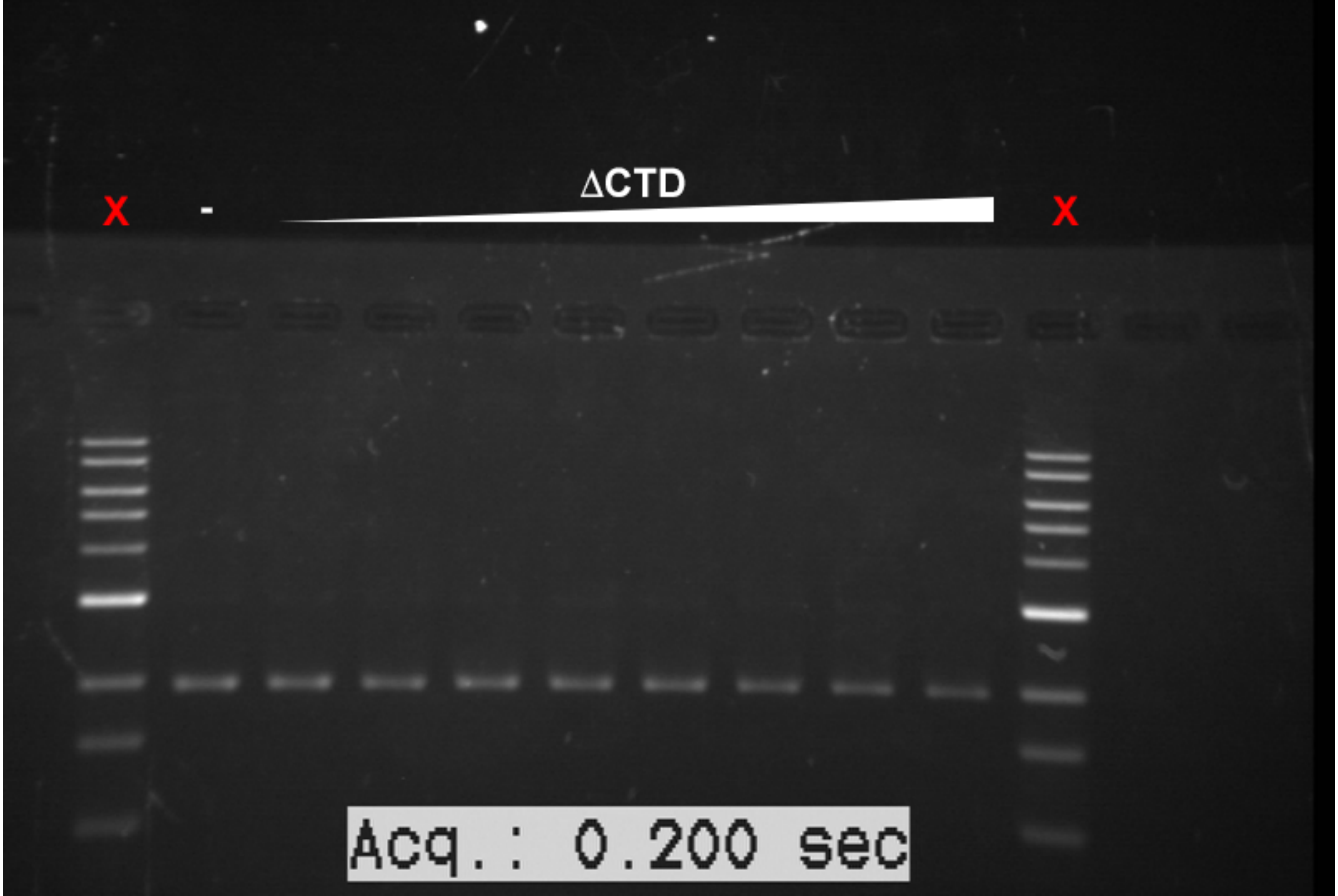

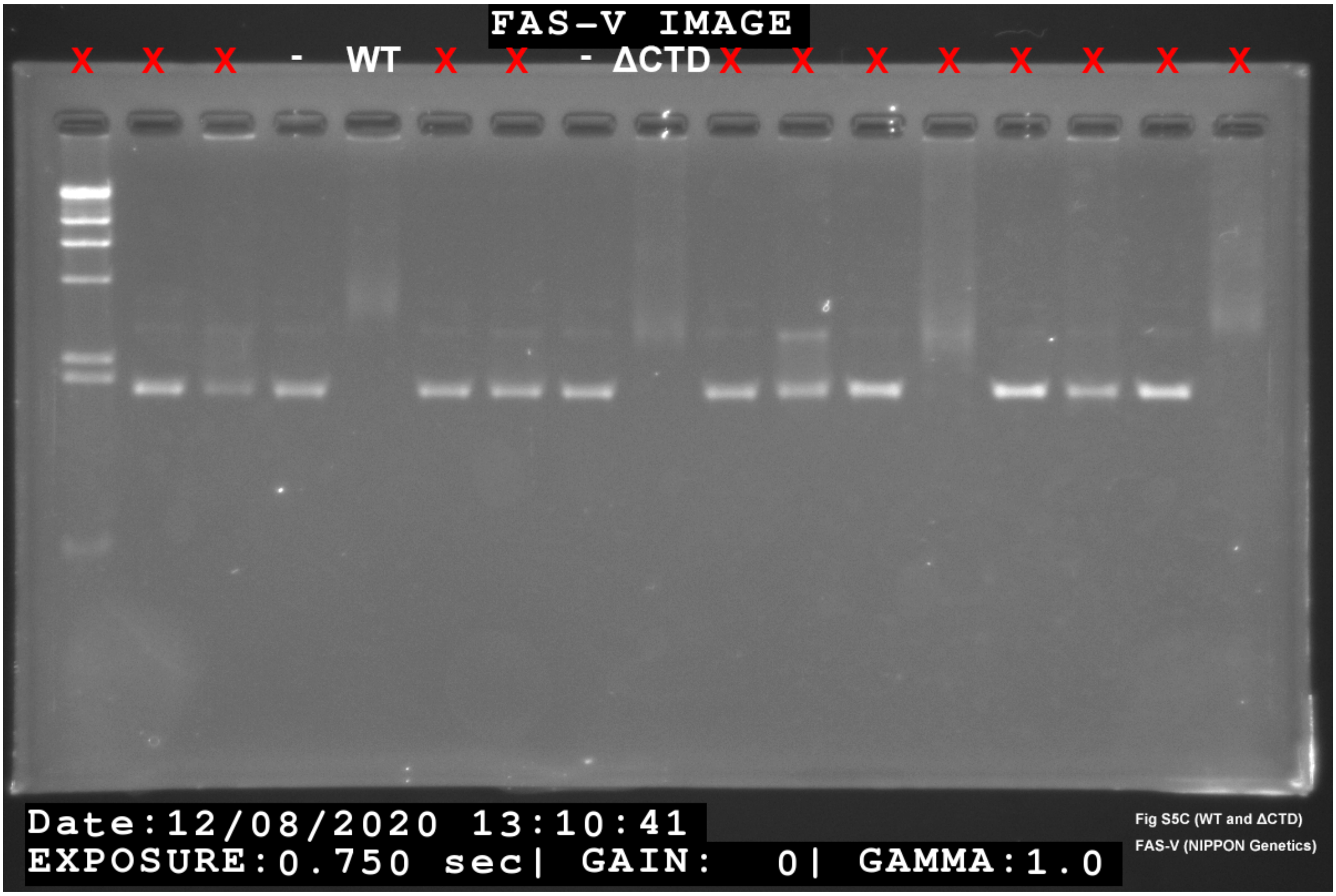
FAS-V IMAGE
X X X - WT X X - ΔCTD X X X X X X X X
Date:12/08/2020 13:10:41
EXPOSURE:0.750 sec| GAIN: 0| GAMMA:1.0
Fig S5C (WT and ΔCTD)
FAS-V (NIPPON Genetics)

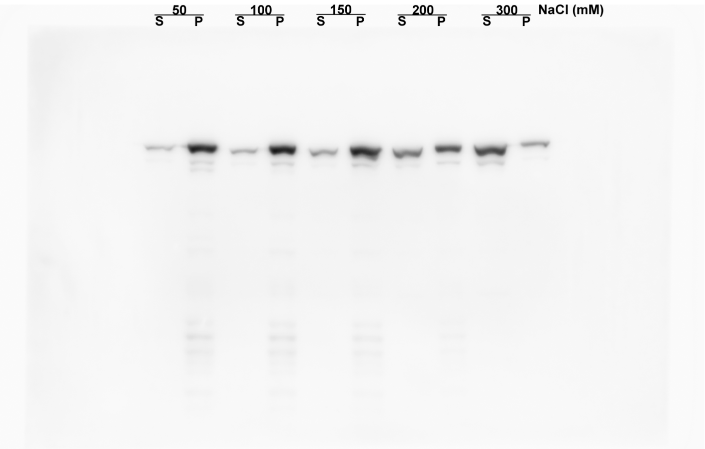

Fig S6 (WT), LAS4000 mini (GE Healthcare)

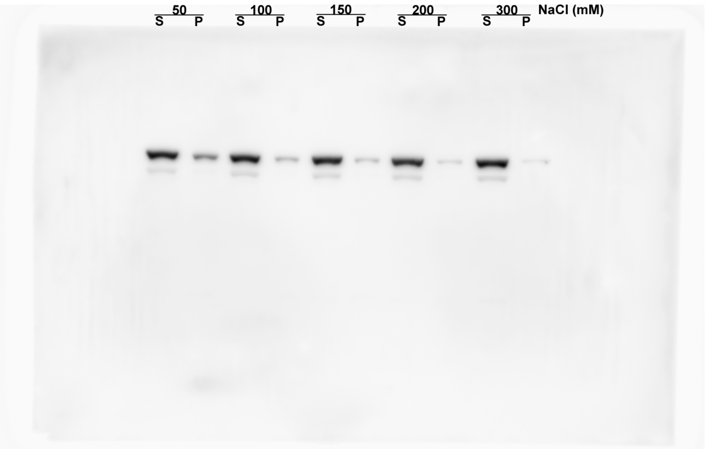

Fig S6 (ΔpCTD), LAS4000 mini (GE Healthcare)
